# XPO1-dependency of DEK::NUP214 leukemia

**DOI:** 10.1038/s41375-025-02570-1

**Published:** 2025-03-27

**Authors:** Fiorella Charles Cano, Arnold Kloos, Rucha Y. Hebalkar, Thomas Plenge, Robert Geffers, Hanna Kirchhoff, Nadine Kattre, Kerstin Görlich, Guntram Büsche, Halyna R. Shcherbata, Michaela Scherr, Konstanze Döhner, Razif Gabdoulline, Michael Heuser

**Affiliations:** 1 Department of Hematology, Hemostasis, Oncology and Stem Cell Transplantation, Hannover Medical School, Hannover, Germany; 2 Institute of Cell Biochemistry, Hannover Medical School, Hannover, Germany; 3 Genome Analytics Research Group, Helmholtz Centre for Infection Research, Braunschweig, Germany; 4 Institute of Pathology, Hannover Medical School, Hannover, Germany; 5 Department of Internal Medicine III, University Hospital of Ulm, Ulm, Germany; 6 University Hospital Halle (Saale), Department of Internal Medicine IV, Martin-Luther-University Halle-Wittenberg, Halle, Germany

**Keywords:** Acute myeloid leukaemia, Targeted therapies

## Abstract

The nuclear export protein XPO1 interacts with nucleoporin 214 (NUP214) and has been implicated in the pathogenesis of SET::NUP214 acute myeloid leukemia (AML). We evaluated DEK::NUP214 (DN), characterizing a distinct AML entity, for its dependency on XPO1 in human AML models. Deletion of XPO1 in DN-positive FKH-1 cells revealed a strong dependency on XPO1. Pharmacologic inhibition of XPO1 by the second-generation selective inhibitor of nuclear export, eltanexor, in primary human and FKH-1 cells reduced XPO1 expression, disrupted co-localization of XPO1 and DN, and induced apoptosis and cell cycle arrest. Functionally, XPO1 and DN co-localized at chromatin, and this co-localization was strongly reduced by XPO1 inhibition. Loss of chromatin binding resulted in downregulation of DN target genes and pathways related to cell cycle and self-renewal. Eltanexor treatment of a patient-derived DN-AML xenograft model disrupted leukemia development, showing molecular clearance in bone marrow after a median of 377 days in eltanexor-treated mice, while control mice succumbed after a median of 244 days. In summary, XPO1 stabilizes DN at chromatin to allow the activation of its oncogenic gene signature, while targeting XPO1 treats leukemia successfully in vivo. These findings establish XPO1 as a molecular target in DEK::NUP214 AML.

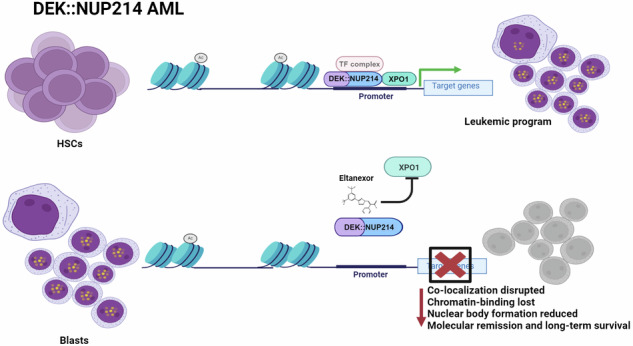

## Introduction

*DEK::NUP214* t(6;9)(p23;q34) acute myeloid leukemia (AML) is recognized as a unique entity by the World Health Organization (WHO) since 2008, as well as the International Consensus Classification (ICC) of hematopoietic neoplasms [1, 2]. It was first identified in 1976 [3], and is found in 1% of patients, mostly affecting younger adults [4]. The breakpoint occurs in the *DEK* gene on chromosome 6 and the *NUP214 gene* (formerly known as *CAN*) on chromosome 9 in defined introns, referred to as icb-6 and icb-9 (intron-containing breakpoints), respectively, leading to the formation of a single chimeric mRNA transcript [5–7]. This group is often co-mutated with the *FLT3* internal tandem duplication (ITD) and characterized by poor prognosis and chemotherapy resistance [8]. Accordingly, the European LeukemiaNet (ELN) assigns patients with DEK::NUP214 to the adverse risk group [9, 10]. The pathogenesis of *DEK::NUP214* AML has been investigated using human CD34+ human hematopoietic progenitor cells transduced with *DEK::NUP214* xenografted into immunocompromised mice as well as syngeneic murine models [11, 12], but models which allow serial transplantation of human AML cells, which would enable pharmacologic testing of new treatment approaches, have not been described so far.

High expression of the *DEK* oncogene in breast and bladder cancers has been associated with advanced disease and poor prognosis [13, 14]. This abundant nuclear protein has been shown to have variable functions in regulation of gene expression, DNA repair, apoptosis, and senescence. The localization of DEK has been shown to be confined to the nucleus and bound to chromatin through the SAP domain and a second DNA-binding structure in the C-terminus. Both DNA binding domains are preserved in the fusion protein [15–18].

The fusion partner NUP214 is a nucleoporin, which is implicated in cell cycle regulation and nucleocytoplasmic transport [19–21]. A previous study found that NUP214 binds to XPO1 (previously known as CRM1), a nuclear export protein involved in the transport of cargos with a nuclear export signal (NES) and RNA species, as part of the nuclear pore complex (NPC) [22]. Phenylalanine-glycine (FG) repeats are found in intrinsically disordered proteins like nucleoporins and localize to the cytoplasmic filaments of the NPC, suggesting these are sites for initial or terminal binding to the transport complex. Specifically, NUP214 is involved in a late step of nuclear export, and it is reported as the nucleoporin with the highest affinity for XPO1 [5, 23]. The DEK::NUP214 fusion protein retains the majority of the FG repeat domains needed for the interaction with XPO1 [24]. XPO1 functions together with the RanGTPase as an energy provider for nuclear transport. An export-independent function of the XPO1/RAN complex has been established in mitosis regulation based on its requirement for microtubule nucleation and centrosome recruitment [25].

This plethora of functions proved to be important in several cancers including hematological malignancies, pancreatic and ovarian cancer, and multiple myeloma [25]. Overexpression of XPO1 is associated with poor prognosis in solid tumor cancers, while single-point mutations have been identified and associated with Hodgkin’s lymphoma and chronic lymphocytic leukemia (CLL) [26]. In AML patient samples, high XPO1 protein levels were established as an independent negative biomarker of overall survival [27].

The clinical use of XPO1 inhibitors like leptomycin B has been complicated by its toxicity [28], resulting in the development of reversible inhibitors like selinexor and eltanexor. As XPO1 has broad functions in physiology and cancer, it was implicated as a general target in many cancers, including AML [29]. Particularly, it was approved in multiple myeloma in combination with bortezomib and dexamethasone for patients after 1–3 prior lines of treatments [30, 31], based on a synergistic effect leading to induction of apoptosis, while it spared normal peripheral blood mononuclear cells, as well as sensitizing myeloma cells to chemotherapy or proteasome inhibitor drugs [32]. However, randomized trials in relapsed/refractory AML did not show an improved survival for selinexor [33–36]. Interestingly, one DEK::NUP214 (DN) patient in a phase I clinical trial with selinexor had a measurable residual disease-negative (MRD) complete remission in response to treatment with this single agent [34, 37, 38], stipulating interest, whether targeting XPO1 may be a rational, targeted treatment in patients with DEK::NUP214 AML. We, therefore, aimed to identify the role of XPO1 in DEK::NUP214-driven human AML.

## Materials and methods

### Cell lines

The human FKH-1 cell line (DSMZ, Braunschweig, Germany), characterized by translocation t(6;9)(p23;q34), was used for all in vitro experiments. The cell lines SEM, OCI-AML2, Kasumi-1, MV4-11 and MOLM-13, which do not not harbor the translocation t(6;9), were used as a negative controls.

### Electroporation and CRISPR/CAS9-mediated XPO1 knockout

FKH-1, OCI-AML2, MV4-11 and Kasumi-1 cells were electroporated with crRNAs [39] (Alt-R® CRISPR-Cas9 crRNA) (sequences described in Supplementary Table S1) targeting the XPO1 gene or a non-targeting sequence in a 3:1 or 2:1 ratio of crRNA-Cas9 (Alt-R™ S.p. Cas9 Nuclease V3) and ribonucleoprotein (RNP) (Integrated DNA Technologies (IDT), Leuven, Belgium). Additional information is provided in Supplementary Methods.

### Patient samples

Frozen bone marrow cells collected during the routine diagnostic workup of patients diagnosed with translocation t(6;9) at the Department of Internal Medicine III at University Hospital of Ulm or Department of Hematology, Hemostasis, Oncology and Stem Cell Transplantation at Hannover Medical School were utilized with informed consent of the patients. All studies were performed in accordance with the Declaration of Helsinki, and the institutional review board of Hannover Medical School (ethical votes 936/2011 and 2504–2014) approved the study. Technical details of in vitro drug experiments are described in Supplementary Methods.

### Patient-derived xenograft models, treatment, and monitoring

All mouse experiments adhered to the guidelines for animal care and use established by Hannover Medical School and were conducted with approval from the Lower Saxony State Office for Consumer Protection, Oldenburg, Germany. Mice were kept under pathogen-free conditions at the central animal laboratory of Hannover Medical School. To establish the patient-derived xenograft (PDX) model female and male animals were used, with age and sex matching to the controls; for drug treatment experiments, 8–11 weeks old female mice were used. Further details are provided in the results and in Supplementary Methods.

### RNA extraction and quantitative RT-PCR

Total RNA was extracted using the RNeasy micro or mini kit according to the manufacturer’s instructions (Qiagen, Düsseldorf, Germany) and reverse transcribed using random primers. Quantitative reverse-transcriptase polymerase chain reaction (RT-PCR) was performed using the Quantitect SYBR green PCR kit (Qiagen) for quantification of double-stranded DNA on a StepOne Plus cycler (Applied Biosystems, Darmstadt, Germany). Relative expression was determined with the 2^−ΔΔCT^ method [40] using *ABL1* as the housekeeping gene to normalize the results. The primer sequences are listed in Supplementary Table S2.

### Bioinformatic analysis of RNA-seq data

Following drug treatment, RNA from FKH-1 and sorted PDX cells was isolated as described above. RNA samples were processed at the Helmholtz Center for Infection Research (Braunschweig, Germany) and sequenced on a Novaseq 6000 instrument (Illumina, San Diego, CA, USA) with an average of 5 × 10^7^ reads per RNA sample. Further information is provided in Supplementary Methods.

### Chromatin immunoprecipitation (ChIP) sequencing

Briefly, 24 h post-drug treatment cells were counted and 20 million cells per condition were washed with ice-cold PBS and resuspended in fixing buffer for 5 min with rotation at room temperature, this was followed by quenching the reaction with glycine for 5 min at room temperature. All buffers contained protease inhibitors (50 µg/ml PMSF, 1 µg/ml leupeptin, and 10 M sodium butyrate). The full description is provided in Supplementary Methods.

### Mutational analysis by next-generation sequencing

A custom TruSight Myeloid Sequencing Panel from Illumina was used to sequence DNA from primary AML patient samples and cells derived from PDX models, following the manufacturer’s instructions and as described before [41, 42] on a MiSeq sequencer (Illumina). This panel includes 48 entire genes or hotspots recurrently found in myeloid leukemias.

### Statistical analysis and figure design

Data are shown as mean ± standard error of mean (SEM). Pairwise comparisons were performed using Student’s *t*-test for continuous variables or one-way ANOVA. The two-sided level of significance was set at *P* < 0.05. Statistically significant results are marked with an asterisk. Survival curves were compared using the log-rank test. Statistical analyzes were performed using Microsoft Excel (Microsoft, Redmond, WA, USA), GraphPad Prism 9 (GraphPad Software, La Jolla, CA, USA), and R (v3.4.4, www.r-project.org). Inkscape v1.2.2 (732a01da63, 2022-12-09) (https://inkscape.org/) was used to create figures. Graphical abstract and Figs. 6A, and S1B, S6A were created with biorender.com

Additional methods such as the clonogenic progenitor assay (CFC), Sanger sequencing, cell cycle analysis, viability and apoptosis staining, co-immunoprecipitation, immunoblotting, morphologic assessment, and immunofluorescence microscopy are described in Supplementary Methods.

## Results

### DEK::NUP214 interacts with and functionally depends on XPO1

**Fig. 1:**
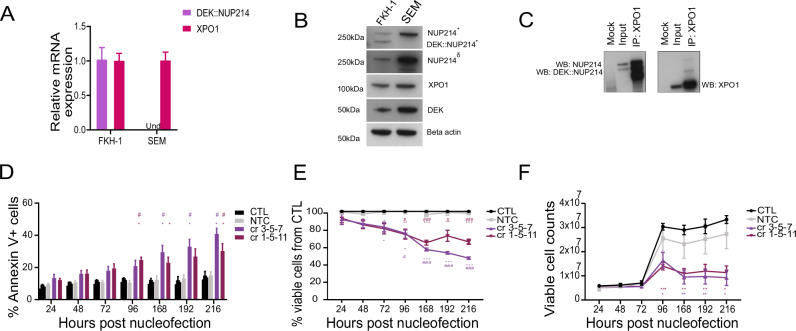
DEK::NUP214 leukemia depends on XPO1*.* **A** Relative mRNA expression of *DEK::NUP214* and *XPO1* in the FKH-1 cell line (mean ± SEM, *n* = 3). The SEM (mean ± SEM, *n* = 3) cell line was used as a control. **B** Western blot showing expression of XPO1, DEK, NUP214, and the DEK::NUP214 fusion protein in FKH-1 and SEM cells. Beta-actin was used as a control. * Western blot using the Bethyl laboratories anti-NUP214 antibody, which recognizes full-length NUP214 and the NUP214 domain preserved in the DEK::NUP214 fusion protein. δ Western blot using the Proteintech anti-NUP214 antibody, which recognizes only full-length NUP214 but not the DEK::NUP214 fusion. **C** Co-immunoprecipitation with western blot evaluating the protein interaction of DEK::NUP214 with XPO1. FKH-1 cells were lysed, and protein extracts were incubated and pulled down with XPO1 antibody. Only magnetic beads were used for mock control. The pull-down was probed with anti-NUP214 (left) and anti-XPO1 (right) antibodiesy and visualized by western blot, 5% of input was used for the input control. **D** Proportion of annexin V+ apoptotic cells in bulk FKH-1 cells after CRISPR RNP electroporation (mean ± SEM, *n* = 3). Control (CTL), non-targeting control (NTC), and CRISPR RNA (cr) targeting the exons of XPO1 are indicated by the numbers in the figure legend. **E** Viability of bulk FKH-1 cells after CRISPR RNP electroporation, assessed as percentage of DAPI negative cells (mean ± SEM, *n* = 3). Control (CTL), Non-targeting control (NTC), CRISPR RNA (cr) targeting the exons of XPO1 indicated by the numbers in the figure legend. **F** Cell counts of FKH-1 cells after CRISPR RNP electroporation (mean ± SEM, *n* = 3). Control (CTL), Non-targeting control (NTC), CRISPR RNA (cr) targeting the exons of XPO1 indicated by the numbers in the figure legend. Statistically significant results are marked with an asterisk or number sign. #*p < 0.05, ##**p < 0.01, ###***p < 0.001. * Mock vs. others, # NTC vs others.

We first validated the expression of the DEK::NUP214 and XPO1 genes and proteins in the FKH-1 cell line, using established leukemia cell lines as the negative control. RT-qPCR and western blot showed that only the FKH-1 cells express the DEK::NUP214 gene and protein, while XPO1 is similarly expressed in the two cell lines (Fig. 1A, B). Sequencing the fusion transcript confirmed the known breakpoint of DEK::NUP214 (Supplementary Fig. S1A) and the interaction of XPO1 with DEK::NUP214 was confirmed by co-immunoprecipitation (Fig. 1C).

To understand the dependency of the DEK::NUP214 fusion protein on XPO1, XPO1 was deleted in FKH-1 cells using CRISPR/CAS9, as well as in control cell lines. A non-targeting sequence (NTC) and a combination of 3 crRNAs, divided into 2 groups, targeting different exons of XPO1 were transfected into FKH-1, OCI-AML2, MV4-11, and Kasumi-1 cells (Supplementary Fig. S1B). Complete uptake was confirmed in all cells targeted with RNPs after nucleofection (Supplementary Fig. S1C).

Initial post-nucleofection stress was observed in cells recovered from all conditions. From 24 h onwards in FKH-1 cells, there was a continuous increase in the percentage of annexin V+ cells in the anti-XPO1 targeted groups, while control and NTC cells remained constant over nine days (Fig. 1D). Consistently, viability decreased rapidly in XPO1 targeted cells compared to control cells (Fig. 1E), while the number of viable cells increased significantly slower in XPO1 targeted cells (Fig. 1F). Reduced expression of the XPO1 transcript and protein was confirmed by RT-qPCR and immunoblotting in anti-XPO1 targeted cells, respectively (Supplementary Fig. S1D, E). Immunofluorescence microscopy was used to characterize the effect of XPO1 depletion on the DEK::NUP214-XPO1 interaction. Control and NTC cells showed typical DEK::NUP214 nuclear bodies and similar expression levels of XPO1 in the nucleus with co-localization of both proteins, while XPO1 targeted cells showed reduced to absent expression of XPO1, loss of co-localization, and a reduced number of DEK::NUP214 nuclear bodies (Supplementary Fig. S1F). XPO1 knockdown was confirmed in control cell lines (Supplementary Fig. S2A), with minimal effects on annexin V+ cells and viability (Supplementary Fig. S2B, C, respectively). Thus, DEK::NUP214 expressing FKH-1 cells depend on XPO1 expression for their oncogenic function.

### Eltanexor effectively inhibits DEK::NUP214 leukemia in vitro

**Fig. 2:**
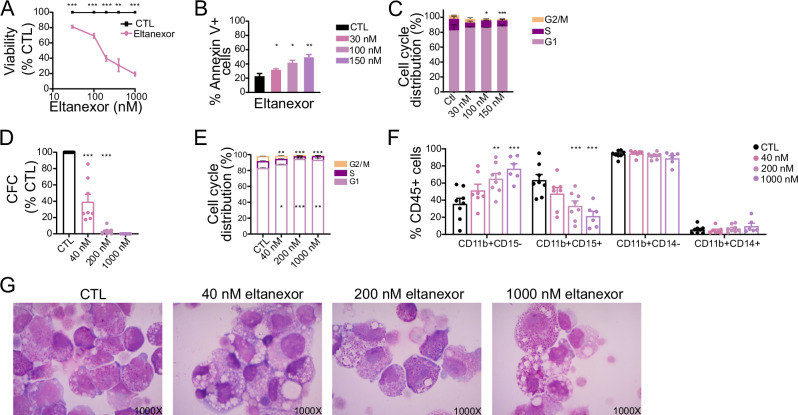
XPO1 can be effectively targeted by XPO1 inhibitor eltanexor in vitro. **A** Cell viability of FKH-1 cells after 96 h of treatment with the indicated concentrations of eltanexor (mean ± SEM, *n* = 3). **B** Frequency of annexin V+ apoptotic FKH-1 cells after 96 h of treatment with the indicated concentrations of eltanexor (mean ± SEM, *n* = 3). **C** Frequency of cell cycle phases in FKH-1 cells after 96 h of treatment with the indicated concentrations of eltanexor (mean ± SEM, *n* = 3). **D** Colony-forming-assay for *DEK::NUP214* positive patient samples. Eight samples were plated in duplicate with the indicated concentrations of eltanexor. Colonies were counted after 10 days of incubation. **E** Frequency of cell cycle phases of *DEK::NUP214* positive patient samples after 72 h of eltanexor treatment (mean ± SEM, *n* = 3). Samples #1, #6, and #8 were used. **F** Immunophenotype of CD45+ cells from *DEK::NUP214* positive patient samples after 72 h of treatment with the indicated concentrations of eltanexor (mean ± SEM, CTL = 8 (samples #1-8), 40 nM, *n* = 7 (samples #1-6, 8); 200 nM, *n* = 8, (samples #1-8); 1000 nM, *n* = 6, (samples #1-2, 4, 6-8). **G** Morphology of a representative *DEK::NUP214* positive patient sample from bone marrow after three days of incubation with eltanexor at the indicated concentrations (original magnification 1000×). Sample #2. Statistically significant results are marked with an asterisk. *p < 0.05, **p < 0.01, ***p < 0.001.

To assess the therapeutic potential of targeting XPO1, the second-generation selective inhibitor of nuclear export (SINE) eltanexor (KPT-8602) was selected for studying the pharmacological inhibition of XPO1. Eltanexor sensitivity was assessed in FKH-1 cells, which had a 50% inhibitory concentration (IC50) of 171.9 nM (Fig. 2A). Treatment with increasing concentrations of eltanexor increased apoptosis (Fig. 2B) and reduced the proportion of cells in S and G2/M phases of cell cycle (Fig. 2C). Moreover, XPO1 protein expression was strongly reduced (Supplementary Fig. S3A), and immunofluorescence microscopy showed a reduction of DEK::NUP214 nuclear bodies (Supplementary Fig. S3B). The first-generation SINE inhibitor selinexor was also evaluated and showed a similar IC50 concentration, induction of apoptosis, and reduction of target gene expression as eltanexor (Supplementary Fig. S4A–C).

The effects of eltanexor were validated in diagnostic bone marrow samples from eight *DEK::NUP214* positive AML patients. All patients were categorized into the ELN adverse risk group due to the presence of the *DEK::NUP214* fusion (Supplementary Table S3) and had been untreated prior to sample harvest.

To assess the clonogenic potential, cells were plated in CFC media supplemented with cytokines and escalating doses of eltanexor. Eltanexor-treated colonies showed rapidly declining colony numbers in a dose-dependent manner when compared to the control (DMSO) (Fig. 2D).

Increasing eltanexor concentrations reduced the viability of primary AML cells in suspension culture (Supplementary Fig. S5A). Cell cycle distribution was also strongly affected by eltanexor resulting in a reduction of the proportion of cells in S and G2/M phases (Fig. 2E). Analysis of the immunophenotype revealed that eltanexor led to an increase of CD11b+/CD15− cells while decreasing the proportion of CD11b+/CD15+ cells (Fig. 2F). These phenotypic changes correlated with morphological analysis of the samples where an increase in monocytic differentiation was observed with more pronounced vacuolization (Fig. 2G). Furthermore, immunofluorescence staining revealed decreased XPO1 protein expression and loss of nuclear bodies upon eltanexor treatment (Supplementary Fig. S5B). These data suggest that inhibition of XPO1 by eltanexor results in cell cycle inhibition and induction of differentiation in DEK::NUP214 primary patient cells.

### XPO1 stabilizes DEK::NUP214 at its chromatin targets

As XPO1 inhibition decreased the DEK::NUP214 nuclear bodies and disrupted the co-localization of both proteins, ChIP-Seq was performed to understand the characteristics of XPO1 and DEK::NUP214 chromatin binding in control and eltanexor-treated cells.

**Fig. 3:**
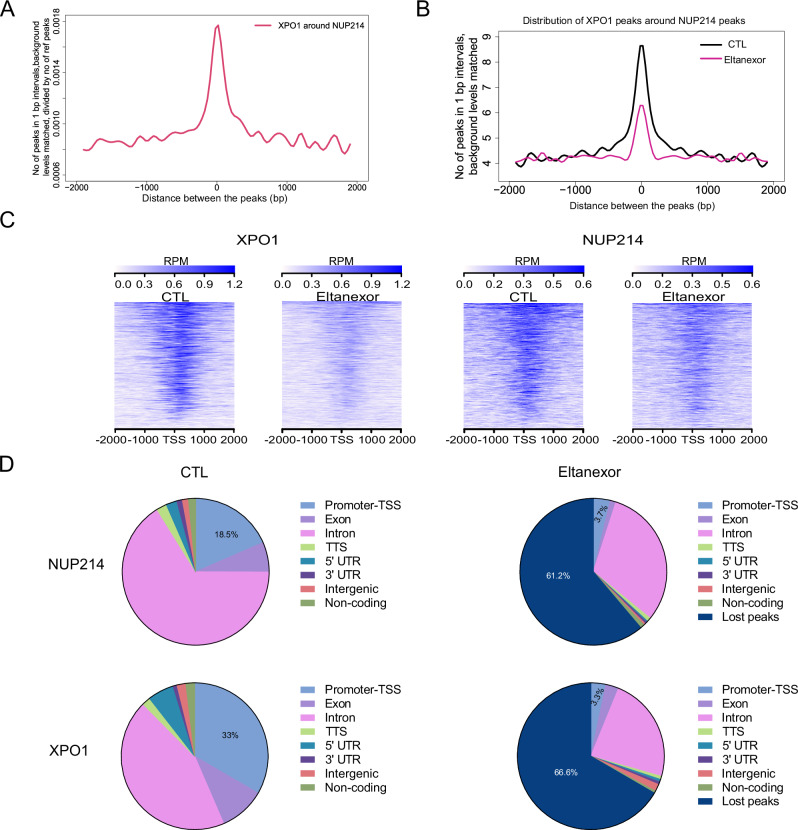
XPO1 recruits DEK::NUP214 to its target genes. **A** Histograms showing the distribution of peaks at XPO1 at NUP214 loci in DMSO-treated FKH-1 cells. **B** Histograms showing the distribution of peaks at XPO1 around NUP214 loci in DMSO and eltanexor treated FKH-1 cells. Aggregation of peak distances calculated at 150 bp bins (12 intervals). **C** Tag density plot of XPO1 and NUP214 chromatin binding surrounding the transcription start site of genes depending on eltanexor treatment. Control (CTL) is DMSO at a 0.1% concentration, as DMSO was used at the same concentration as solvent for eltanexor. **D** Distribution of genomic elements associated with XPO1 and NUP214 chromatin binding across the 8000 genes commonly bound in FKH-1 CTL and eltanexor treated cells. The total number of chromatin peaks of NUP214 in control treated cells is 3148 peaks, and of NUP214 in eltanexor treated cells is 1222 peaks. The total number of chromatin peaks of XPO1 in control treated cells is 12,836 peaks, and of XPO1 in eltanexor treated cells is 4285 peaks. TSS transcription start site, TTS transcription termination site, UTR untranslated region.

For XPO1 and DEK::NUP214 control-treated cells, 17,541 and 4928 chromatin loci were observed, respectively. Aggregation of peak distances of XPO1 loci around NUP214 binding sites showed strong enrichment of XPO1 at NUP214 sites (Fig. 3A). Upon eltanexor treatment, the enrichment of XPO1 peaks around NUP214 peaks decreased (Fig. 3B).

Analysis of XPO1 and NUP214 binding sites within ±1000 bp of the transcription start sites of known genes indicated that ~8000 genes had at least one peak of either protein within their transcription start site (TSS). Moreover, the global decrease of XPO1 and NUP214 chromatin binding after eltanexor treatment was confirmed by a 2.8 and 1.4-fold reduction of chromatin peaks of XPO1 and NUP214, respectively (Fig. 3C). Additionally, the relative distribution of these peaks across the gene structure was studied to better understand the dynamics of chromatin binding. In control samples, 18.5% and 33% of NUP214 and XPO1 peaks were located in the promoter-TSS, respectively. Eltanexor treatment reduced these peaks to 3.7% and 3.3% (Fig. 3D), suggesting that DEK::NUP214 requires XPO1 for the execution of its leukemogenic transcriptional program.

### XPO1 inhibition leads to downregulation of putative DEK::NUP214 target genes

Since the loss of the XPO1 protein has profound effects on viability and cell cycle in DEK::NUP214 expressing cells and leads to loss of promoter binding, we next evaluated the transcriptional consequences of eltanexor treatment.

**Fig. 4:**
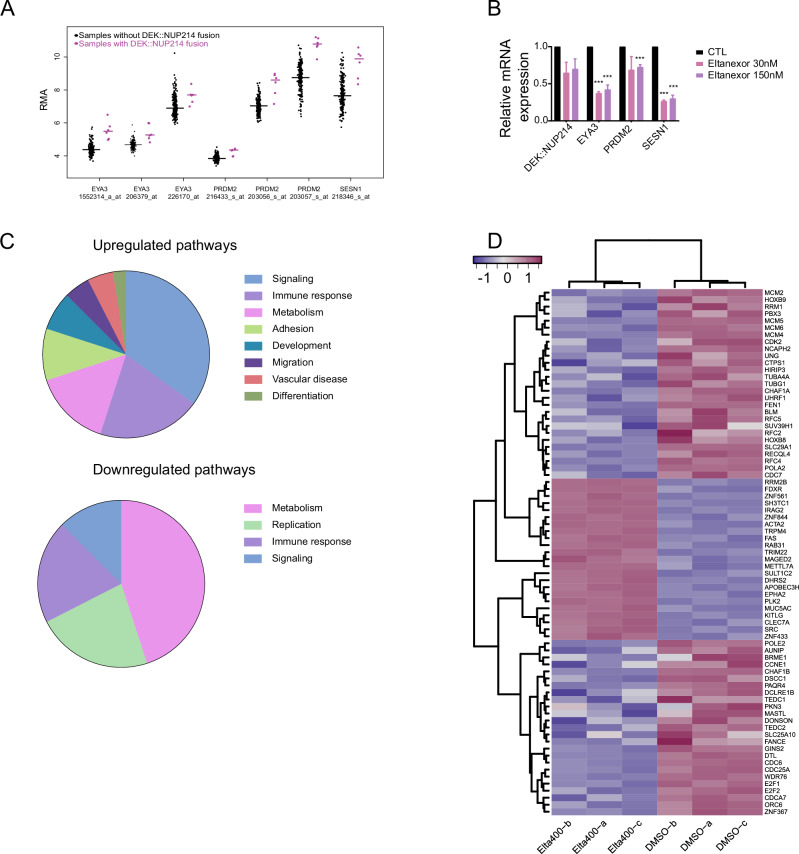
XPO1 inhibition leads to downregulation of putative DEK::NUP214 target genes. **A** Gene expression of putative DEK::NUP214 target genes in *DEK::NUP214* positive and *DEK-NUP214* negative AML patients, based on published gene expression data (GSE17855); (DN patients = 6, purple, other leukemia = 231, black, EYA3 probes 1552314_a_at, *p* = 0.0038, 206379_at *p* = 0.0144, 226170_at *p* = 0.0220; PRDM2 probes 203056_s_at *p* = 0.0057, 203057_s_at *p* = 0.0070, 216433_s_at *p* = 0.00008; SESN1 probe, 218346_s_at *p* = 0.0035). **B** Changes in gene expression of *DEK::NUP214* putative target genes in FKH-1 cells after 96 h of treatment with eltanexor at the indicated concentrations (mean ± SEM, *n* = 3). **C** Heatmap from unsupervised hierarchical clustering showing the top 100 differentially expressed genes in FKH-1 cells after 24 h of treatment with eltanexor at a concentration of 400 nM or solvent control (DMSO 0.1%) (*n* = 6). **D** Gene set enrichment analysis for FKH-1 cells treated with eltanexor at a concentration of 400 nM for 24 h or solvent control-based KEGG pathways. Statistically significant results are marked with an asterisk. *p < 0.05, **p < 0.01, ***p < 0.001.

Analysis of the publicly available gene expression profiles of 6 DEK::NUP214 patients and 231 AML patients with newly diagnosed pediatric AML without the DEK::NUP214 fusion revealed a unique gene signature for this leukemia subtype as demonstrated by Sandahl et al. [8]. Particularly, EYA3, PRDM2, and SESN1 were highly expressed in DEK::NUP214 positive patients (Fig. 4A). We further confirmed this by analyzing the Beat AML database that contains 3 DEK::NUP214 patients and 448 AML patients diagnosed with other subtypes, where this pattern of upregulation was observed as well (EYA3, *p* = 0.005, PRDM2, *p* = 0.024, SESN1, *p* = 0.013, data not shown). Using RT-qPCR in eltanexor or control-treated FKH-1 cells we show that these putative target genes are significantly downregulated by eltanexor (Fig. 4B) and selinexor (Supplementary Fig. 4C). In addition, reduced chromatin binding of XPO1 and NUP214/DEK::NUP214 was observed on these genes (Supplementary Fig. S6A). The eltanexor-induced differential gene expression pattern was further evaluated by gene set enrichment analysis (GSEA, Fig. 4C). Among the top 30 enriched gene sets comparing eltanexor with vehicle-treated cells, the majority of gene sets related to signaling and immune response (Fig. 4C and Supplementary Table S4). Among the most downregulated genes were HOX genes (e.g., HOXB8, HOXB9, PBX3) and cell cycle regulators (e.g., E2F1, CDC7, Fig. 4D and Supplementary Table S5). The top downregulated gene sets involved processes of metabolism and replication, supporting that eltanexor induces differentiation and cell cycle inhibition (Fig. 4D).

### DEK::NUP214 primary AML cells engraft in PDX mice and are serially transplantable

To validate the effect of eltanexor on DEK::NUP214 leukemia in vivo, we developed a serially transplantable PDX model of DEK::NUP214 AML.

**Fig. 5:**
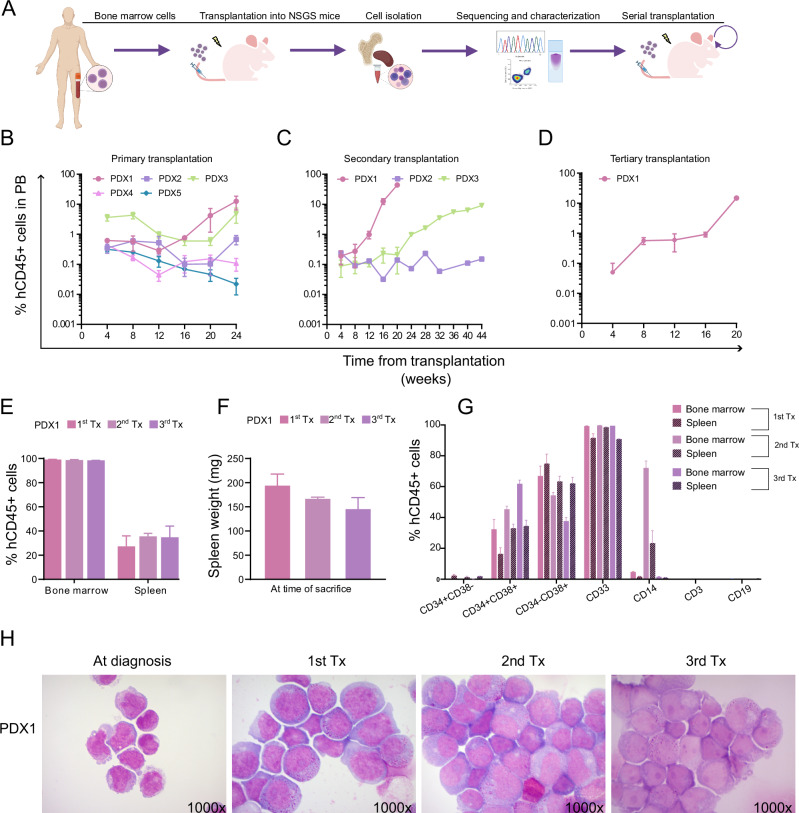
Development of PDX models. **A** Outline for the development of a patient-derived xenograft (PDX) model from *DEK::NUP214* fusion-positive patients. Engraftment kinetics of human CD45+ cells in peripheral blood from PDX models at the indicated time points across the first (**B**), second (**C**), and third (**D**) transplantation (1st, 2nd Tx, mean ± SEM, n = 3, 3rdTx mean = 2). **E** Engraftment of human CD45+ cells in bone marrow and spleen at the time of sacrifice (mice of first and second transplants were sacrificed after 24 weeks (mean ± SEM, *n* = 3), mice of the third transplant were sacrificed after 20 weeks (3rdTx mean = 2)). **F** Spleen weight in mice of model PDX1 at time of sacrifice after the first, second, and third transplantation (1st, 2ndTx, mean ± SEM, *n* = 3, 3rdTx mean = 2). **G** Immunophenotype of human CD45+ cells from PDX1 in bone marrow and spleen at the time of sacrifice after the first, second, and third transplantation (see **F**). **H** Morphology of bone marrow cells at diagnosis of the DEK::NUP214 positive patient (left), whose cells gave rise to PDX1, and of bone marrow cells after samples after the first, second, and third transplantation of PDX1 (original magnification 1000×). Tx transplantation.

Five bone marrow samples of DEK::NUP214 positive AML patients were transplanted by intravenous injection in the tail vein of immunocompromised NSGS mice (Fig. 5A). Engraftment of human cells was measured by the hCD45+ fraction in peripheral blood of mice, quantified once a month.

Twenty-four weeks post-transplantation, three patient samples showed a mean of 12.53 (PDX1), 0.68 (PDX2), and 4.89 (PDX3) percent hCD45+ cells in peripheral blood (Fig. 5B). These mice were then sacrificed, and a mixture of bone marrow and spleen cells were transplanted into secondary recipients. Secondary recipients of PDX1 cells showed a rapid increase in hCD45+ cells at week 16. These cells were then transplanted into tertiary recipients. PDX2 did not engraft in secondary recipients over a 44 weeks period and no additional transplantation was performed. One mouse of the PDX3 recipients showed detectable engraftment at week 28 and was sacrificed for analysis at week 44, while a second mouse was sacrificed with no engraftment at week 24 (Fig. 5C). PDX1 tertiary recipients showed faster engraftment kinetics when compared with initial transplantations (Fig. 5D).

Each transplantation of PDX1 cells was characterized by expression of cell surface markers in peripheral blood, bone marrow, and spleen at the time of sacrifice. PDX1 had >99% engraftment of hCD45+ cells in bone marrow, whereas in the spleen it increased from first to second transplantation (Fig. 5E). At time of sacrifice spleens were enlarged (Fig. 5F).

Next, we evaluated the stem cell and progenitor compartments in bone marrow and spleen of PDX1 mice during serial transplantation. For bone marrow cells, the CD34+CD38− population accounted for less than 1%. Early progenitors (CD34+CD38+) increased from 32% to 61%, while lineage-committed progenitors (CD34-CD38+) decreased from 69% to 37%. Other lineage markers were detected at low levels except for CD14 (Fig. 5G, filled bars). Similar patterns were observed in spleen cells (Fig. 5G, black diagonal filled bars). PDX1 cells showed a similar immunophenotype compared to the patient’s blasts (data not shown).

Genetic analysis of PDX1 cells showed that all mutations diagnosed in the patient sample were retained across transplantations (Supplementary Table S6). Morphologic analysis confirmed a similar blast phenotype of PDX1 cells compared to the patients’ cells with partially granulated cytoplasm (Fig. 5H). Thus, primary DEK::NUP214 + AML cells could be engrafted and serially transplanted in immunodeficient mice and maintained the genetic and phenotypic characteristics of the primary cells.

### DEK::NUP214 AML is exceptionally sensitive to XPO1 inhibition in vivo

**Fig. 6:**
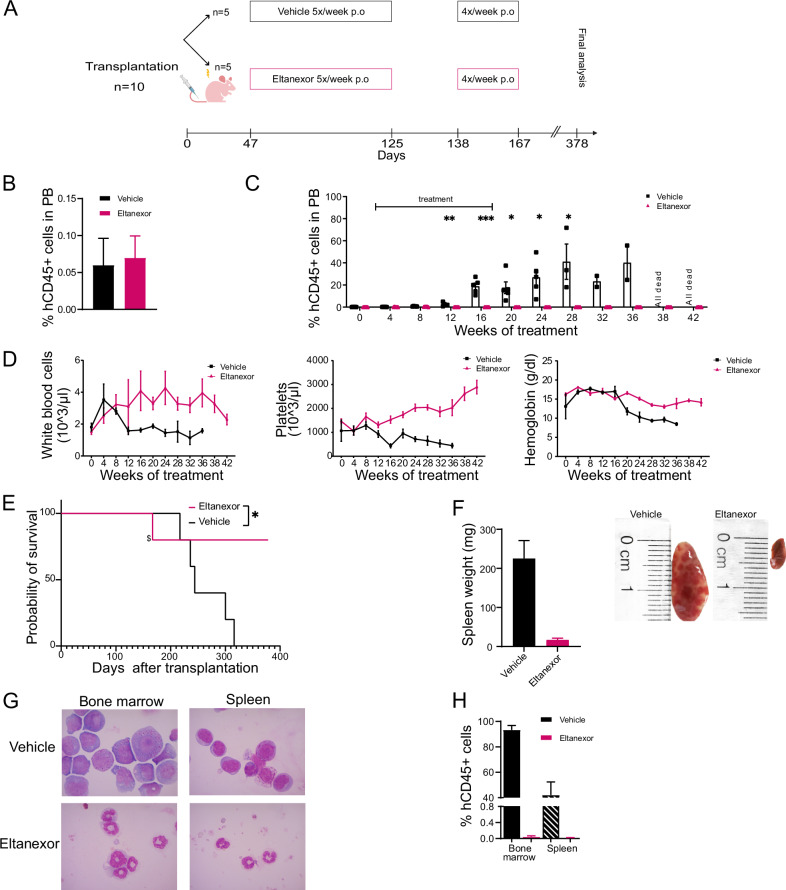
DEK::NUP214 leukemia depends on XPO1 in vivo. **A** Treatment outline for in vivo treatment of the DEK::NUP214 positive PDX1 model with eltanexor. Six weeks after transplantation, mice received 12.5 mg/kg eltanexor once daily for 5 days per week for 12 weeks, and during the second treatment cycle 10 mg/kg eltanexor once daily for 4 days per week for 4 weeks. **B** Engraftment of human CD45^+^ cells in peripheral blood before the start of treatment (mean ± SEM, *n* = 5, per group). **C** Engraftment of human CD45^+^ cells in peripheral blood in vehicle and eltanexor treated mice (mean ± SEM, *n* = 5, per group). Week 20, 4 mice for eltanexor group, week 28, 3 mice for vehicle group, week 36 2 mice for vehicle group. **D** White blood cell count, platelet count, and hemoglobin during the duration of the experiment in vehicle and eltanexor treated mice (mean ± SEM, *n* = 5, per group). Week 20, 4 mice for eltanexor group, week 28, 3 mice for vehicle group, week 36 2 mice for vehicle group. **E** Survival of DEK::NUP214 positive AML PDX1 mice treated with vehicle or eltanexor (*n* = 10). $ mouse was sacrificed due to weight loss, no engraftment in peripheral bood, spleen, or bone marrow was observed. **F** Spleen weight of vehicle and eltanexor treated mice at time of sacrifice (mean ± SEM, *n* = 5, per group). Representative images of the spleen from one vehicle and eltanexor-treated mouse at time of sacrifice. The image background was removed for clarity. **G** Representative images of cytospin preparations of bone marrow and spleen cells from vehicle and eltanexor-treated mice at time of sacrifice. **H** Engraftment of human CD45^+^ cells in bone marrow and spleen of vehicle and eltanexor-treated mice at time of sacrifice (mean ± SEM, *n* = 5). Statistically significant results are marked with an asterisk. *p < 0.05, **p < 0.01, ***p < 0.001.

Due to the specific dependency of DEK::NUP214 cells on XPO1 we evaluated eltanexor in our PDX1 model. The experimental setup and treatment schedule are shown in Fig. 6A.

Thirty-five days post-transplantation; mice were randomized into two groups to receive vehicle or eltanexor, respectively. Both groups had similar leukemic engraftment at this time (Fig. 6B). The first treatment cycle with eltanexor (12.5 mg/kg p.o., 5 days/week) or vehicle was given from day 47 to day 125 after transplantation. Treatment was interrupted from day 126 to day 135 days and then resumed at a dose of 10 mg/kg for 4 days/week from day 138 to day 167. Mice were monitored monthly by peripheral blood assessment for engraftment of hCD45+ cells. In control mice, engraftment increased from 1% to 40% over 38 weeks, when the last moribund mouse was sacrificed. In contrast, no hCD45+ cells could be detected in peripheral blood in eltanexor-treated mice throughout the duration of treatment, and an additional 26 weeks after the last control mouse had been sacrificed (Fig. 6C). Over time WBC, platelets, and hemoglobin decreased in control mice, while eltanexor-treated mice retained normal blood counts (Fig. 6D).

Importantly, the eltanexor-treated mice showed a significant survival advantage compared to the control mice (median survival not reached vs 244 days after transplantation, *p* = 0.0034) (Fig. 6E). One eltanexor-treated mouse had to be sacrificed at 167 days due to weight loss, possibly related to drug treatment. No human CD45+ cells were detected in peripheral blood, bone marrow and spleen of this mouse suggesting eradication of the disease at this time.

At the time of sacrifice control mice showed enlarged spleens, while eltanexor-treated mice had regular sized spleens (Fig. 6F). Morphology from cytospin preparations of bone marrow and spleen confirmed blast infiltration in control mice, while no blasts were detectable in eltanexor treated mice (Fig. 6G). Immunophenotyping of control-treated mice showed a mean engraftment of hCD45+ cells in bone marrow and spleen of 93.2% and 42.1%, respectively, while no hCD45+ cells could be identified in eltanexor-treated mice (Fig. 6H). Bone marrow and spleen cells of control-treated mice had a similar immunophenotype as the previous transplantations of PDX1 cells (Supplementary Fig. S7A). RT-qPCR confirmed the expression of the DEK::NUP214 fusion gene in bone marrow of sacrificed control mice but could not detect the fusion gene in bone marrow of eltanexor-treated mice (data not shown).

Differentially expressed genes were evaluated in sorted hCD45+PDX1 cells from bone marrow after an 11-day treatment period. The most downregulated genes in eltanexor treated mice included HOX and cell cycle genes as in the in vitro treated FKH-1 cells (Supplementary Table S7). PCA analysis demonstrated similarities between the FKH-1 cell line and the PDX1 model following eltanexor treatment (Supplementary Fig. S7B), while GSEA revealed common pathways, such as enriched immune response and metabolism (Supplementary Fig. S7C, Supplementary Tables S8, S9, S10).

Due to the co-occurrence of *FLT3* mutations in DEK::NUP214 patients, we examined the potential synergy of eltanexor and gilteritinib, an FTL3 inhibitor, in five leukemia cell lines (Supplementary Table S12 and Supplementary Fig. S8). We identified that combination treatment in FKH-1 cells at high doses leads to a synergistic effect.

In summary, XPO1 inhibition by eltanexor eradicated primary human DEK::NUP214 leukemia cells in vivo and proved an exceptional on-target sensitivity of these cells in a primary human AML model.

## Discussion

Functional investigation of the role of XPO1 in DEK::NUP214 AML showed that the oncogenic function of DEK::NUP214 depends on XPO1, as deletion of XPO1 led to cell cycle arrest and apoptosis in vitro, while pharmacologic inhibition of XPO1 by eltanexor cured PDX mice from DEK::NUP214 AML. Inhibition of XPO1 strongly reduced chromatin binding of both XPO1 and NUP214, downregulated DEK::NUP214 target genes and pathways related to cell cycle and metabolism, and disrupted nuclear body formation. These findings establish XPO1 as a molecular target in DEK::NUP214 AML.

NUP214 proteins are frequent partners in chromosomal translocations like NUP214::ABL1, SQSTM1::NUP214, SET::NUP214, and DEK::NUP214, which can occur in AML and ALL [19, 21]. The NUP214 protein is located in the periphery of the nuclear pore complex (NPC), where it mediates nuclear traffic and maintains barrier permeability. NUPs have also been implicated in chromatin reorganization and gene regulation by chromatin binding, both in a nuclear-transport dependent and independent manner [43–46]. The oncogenic function of NUP214 fusion proteins has been attributed to their re-localization from the cytoplasmatic filaments of the NPC to the nucleus, their interaction with XPO1 [24], activation of STAT- and mTOR- signaling, and inhibition of NF-kB-transcriptional activation [47–49].

The wild-type DEK protein is an epigenetic regulator, which is characterized by two DNA binding domains and histone binding [50]. The DN fusion protein contains almost the full-length DEK sequence and interferes with wild-type DEK, leading to loss of the interaction with known interacting proteins like CK2 and histone H3 [51]. Our results show that XPO1 and DN co-occupy chromatin, and that XPO1 is required to maintain binding of DN to DNA. In a NUP98-HOXA9 fusion model it was shown that chromatin binding of the fusion protein was dependent on pre-bound XPO1. Chromatin binding of XPO1 has also been shown in NPM1c and SET::NUP214 leukemia [52], suggesting that XPO1 mediates and stabilizes chromatin binding of DN [53].

We evaluated the role of XPO1 by genetic deletion and pharmacologic inhibition. Eltanexor led to a strong reduction of XPO1 protein levels, imitating the genetic deletion induced by CRISPR/CAS9. It has been described previously that binding of SINEs to the NES groove of XPO1 leads to conformational changes that mark the protein for proteasomal degradation by E3 ubiquitin ligases [54–56]. This suggests that loss of the XPO1 protein mediates the observed effects.

The formation of nuclear bodies in NUP fusions has been shown to co-localize with XPO1 and regulate aberrant gene activation [57, 58]. Nuclear bodies are involved in spatial organization of the nucleus, supporting compartmentalization [59]. In our study, both the FKH-1 cell line and primary AML cells were characterized by nuclear bodies co-localizing with XPO1 and DN, which disappeared upon eltanexor treatment and genetic deletion of XPO1. Saito et al. [60] showed that the formation of nuclear bodies can dysregulate XPO1-mediated nuclear export in SET::NUP214 expressing cells. We observed that eltanexor treatment downregulated cell cycle and self-renewal associated gene sets, while metabolism pathways were dysregulated. It is thus likely that XPO1 mediates the oncogenic effects of DN by dysregulation of transport and cell cycle on protein level besides its effects on transcription.

We selected eltanexor over selinexor due to its improved safety profile, likely due to its reduced crossing of the blood-brain barrier [61]. A prior study in *NPM1* mutant leukemia models also showed sensitivity to SINE compounds, while the treatment prolonged survival by approximately 24–25 days, but could not eradicate the disease [62]. This supports a high specificity of eltanexor for DN AML and a critical vulnerability of DN disease.

XPO1 inhibition by selinexor is approved for second-line treatment of multiple myeloma [63]. However, a randomized study in relapsed/refractory AML patients comparing selinexor with the best available therapy did not show an improved survival with selinexor [34]. Interestingly, one DEK::NUP214 AML patient in a phase I clinical trial with selinexor as a single agent had an MRD-negative complete remission [34, 37, 38], underscoring the clinical potential of our findings.

The synergistic effect observed in the FKH-1 cells could be related to the upregulation of AXL, another target of gilteritinib [64, 65]. This upregulation was observed after 24 h treatment with eltanexor (Supplementary Table S5). It has been shown that dual inhibition of both kinases can lead to improved targeting of the bone marrow niche [66]. In addition, a significant proportion of FLT3 wildtype patients respond to FLT3 inhibitors, likely due to an FLT3-like gene expression signature, found in the absence of FLT3 mutations [67–74].

For DEK::NUP214, one patient was reported where gilteritinib was used after failure of standard induction chemotherapy. This patient achieved complete remission after eight weeks of treatment [75]. It is worth noting that sorafenib in combination with azacitidine was also able to induce remission in a DEK::NUP214 patient [76]. However, another study showed that seven patients positive for DEK::NUP214 who had relapsed were treated with lestaurtinib, gilteritinib or sorafenib, alone or in combination with standard chemotherapy, and failed to respond. One patient who received gilteritinib monotherapy achieved complete remission [77]. These data suggest that XPO1 and FLT3 are co-dependent in DEK::NUP214 patients and their combined targeting may enhance treatment response and outcome. On the other hand, the SINE inhibitors may be combined with standard treatments for AML patients like intensive chemotherapy [78] or a hypomethylating agent with venetoclax [79], which showed synergistic effects with selinexor and eltanexor in vitro [80].

In summary, we propose XPO1 as a molecular target in DEK::NUP214 AML, which is required for chromatin binding of DEK::NUP214, nuclear body formation, and activation of the leukemic program of DEK::NUP214. Based on the induction of molecular remission and cure of DEK::NUP214 human AML in a PDX model by single agent eltanexor we suggest that this strategy should be evaluated in DEK::NUP214 AML patients.

## Supplementary information

Supplementary files

Supplementary Table S4: Gene set enrichment analysis of gene expression profiles from FKH-1 eltanexor treated vs FKH-1 control treated cells

Supplementary Table S5: Top500 differentially expressed genes in FKH-1 samples

Supplementary Table S7: Top 500 differentially expressed genes in hCD45+ bone marrow cells from PDX1 mice after 11 days of treatment with eltanexor or vehicle

Supplementary Table S8: Gene set enrichment analysis of gene expression profiles from PDX1 eltanexor treated vs PDX1 vehicle treated cells

Supplementary Table S9: Comparison of common upregulated pathways between eltanexor treated PDX1 and FKH-1 cells compared to control treated PDX1 and FKH-1 cells

Supplementary Table S10: Comparison of common downregulated pathways between eltanexor treated PDX1 and FKH-1 cells vs control treated PDX1 and FKH-1 cells

## Data Availability

RNA-seq and ChIP-seq data are available at Gene Expression Omnibus (GEO) under accession numbers GSE270396 and GSE270397, respectively.
