# Supplementary material for: XPO1-dependency of DEK::NUP214 leukemia
Source: Leukemia. 2025 Mar 27;39(5):1102–13. doi: 10.1038/s41375-025-02570-1 (PMC12055596; doi:10.1038/s41375-025-02570-1)
Supplement: Supplementary file 1 — Supplementary files [file 41375_2025_2570_MOESM1_ESM.docx]

**XPO1-dependency of DEK::NUP214 leukemia**

Fiorella Charles Cano^1^, Arnold Kloos^1^, Rucha Y. Hebalkar^2^, Thomas Plenge^1^, Robert Geffers^3^, Hanna Kirchhoff^1^, Nadine Kattre^1^, Kerstin Görlich^1^, Guntram Büsche^4^, Halyna R. Shcherbata^2^, Michaela Scherr^1^, Konstanze Döhner^5^, Razif Gabdoulline^1^, Michael Heuser^1,6^

**Supplemental Information**

**Supplemental Materials and Methods**

**Cell culture**

Cells were cultured in RPMI 1640 medium, IMDM or alpha-MEM (Gibco, Thermofisher, Waltham, MA), supplemented with fetal calf serum (20% or 10%, FCS) (Sigma-Aldrich, Taufkirchen, Germany), 1% penicilin-streptomycin (Gibco, Thermofisher) and a prophylactic dose of plasmocin® (Invivogen, Toulouse, France) at 37°C and 5% CO_2_. For all cell lines, expression of the fusion gene was confirmed by RT-qPCR as described below.

**Electroporation and CRISPR/CAS9-mediated XPO1 knockout**

crRNA and tracrRNA-ATTO550 (Alt-R® CRISPR-Cas9 tracrRNA, ATTO™ 550, Integrated DNA Technologies (IDT), Leuven, Belgium) were mixed in equimolar concentrations to form a final duplex. This duplex was incubated at 95°C for 5 min and allowed to cool down to room temperature, followed by preparation of ribonucleoprotein complexes and incubation for 20 minutes at room temperature.

FKH-1 and MV4-11 cells were prepared for electroporation using the cell nucleofection kit L (Lonza, Basel, Switzerland), program Q-001 using the Amaxa nucleofector device 2b (Lonza), according to the manufacturer’s instructions, including the electroporation enhancer (IDT). OCI-AML2 and Kasumi-1 cells were electroporated using nucleofection kit V, program X-001 and P-19, respectively. A mock control electroporation was included. Electroporation efficiency was evaluated by flow cytometry (Cytoflex S, Beckman Coulter Life Sciences, Krefeld, Germany).

**Drug assays**

Cell lines were treated with KPT-802, selinexor, gilteritinib, alone or in combination for 72 to 96 hours in vitro. The compounds were purchased from Selleckchem (Frankfurt, Germany). Synergy calculations were perfomed using the software CompuSyn 1.0 (ComboSyn Inc., Paramus, NJ).

**Viability and apoptosis staining**

Cells were washed twice with PBS and resuspended in 1X binding buffer according to manufacturer’s instructions (BD Biosciences, Heidelberg, Germany) with AnnexinV-APC and DAPI. Subsequently, cells were incubated for 20 minutes in the dark and analysed with the Cytoflex S cytometer (Beckman Coulter).

**Patient-derived xenograft models, treatment and monitoring**

One million primary patient cells or freshly isolated patient-derived xenograft (PDX) cells were transplanted intravenously in the lateral vein of sublethally irradiated (2.5 Gy) NOD.Cg-Prkdcscid.Il2rgtm1Wjl/SzJ mice transgenic for human interleukin-3, granulocyte-macrophage-colony-stimulating factor and stem cell factor (NSGS). If deemed necessary samples were depleted of CD3+ cells with CD3 microbeads (Miltenyi Biotec, Bergisch Gladbach, Germany) according to manufacturer’s instructions. At time of sacrifice, spleens from each mouse were imaged and the image background was removed for clarity using Adobe Photoshop 2024 (Adobe, San Jose, CA). The size of the animal cohorts was based on the amount of viable cells available for transplantation. No animals were excluded during the experiments and **blinding was not used in animal experiments since all animal experiments were performed with a homogeneous strain, age, and similar variance.**

**Cell culture of AML patient cells**

For in vitro studies, primary human AML samples were thawed and stimulated 24 hours in Iscove's Modified Dulbecco's Medium (IMDM) (STEMCELL Technologies Inc, Vancouver, Canada) supplemented with 20% bovine serum albumin, insulin and transferrin (BIT 9500, STEMCELL Technologies), 100 µM 2-mercaptoethanol (Sigma-Aldrich), 2 mM L-Glutamine (Gibco), 100 ng/ml human FLT3-ligand, 100 ng/ml human stem cell factor (SCF), 20 ng/ml human IL-3, 20 ng/ml human IL-6, 20 ng/ml human granulocyte colony-stimulating factor (G-SCF), 50 ng/ml human thombopoietin (TPO) (all from PeproTech, Hamburg, Germany), and 1 % penicilin-streptomycin (Gibco) before drug treatment or clonogenic progenitor assays.

**Clonogenic progenitor assay**

Colony-forming cells (CFCs) were assayed in methylcellulose (Methocult H4100, StemCell Technologies) supplemented with 10 ng/mL human IL3, 10 ng/mL human GM-CSF, 50 ng/mL human SCF, 50 ng/mL human FLT3-ligand and 3 U/mL human EPO (Peprotech). Vehicle (DMSO) or eltanexor (1000, 200, 40 nM) was added to 50,000 cells, which were plated in duplicates. Colonies were evaluated microscopically 10 days after plating by standard criteria.

**RT- PCR and Sanger sequencing**

Reverse transcribed RNA was used to amplify a 500 bp product of the DEK::NUP214 fusion transcript containing the breakpoint. The PCR product was purified with Qiagen PCR purification kit according to manufacturer’s instructions. The PCR product was then Sanger sequenced by Eurofins Genomics GmbH (Konstanz, Germany) and the BLAST® tool was used to confirm the fusion gene. The chromatogram was visualized with Teal (https://www.gear-genomics.com/teal/). [1] Primer sequences are listed in Supplemental Table S2.

**Cell cycle analysis**

Cells were washed twice with PBS and fixed with ice-cold 70% ethanol for 2 hours. After washing, cells were resuspended in PBS with 10 µg/ml DAPI/Triton X-100 and analysed with the Cytoflex S (Beckman Coulter). The cycle phase was determined based on the intensity; DAPI^low^ cells represent cells in the G1 phase, DAPI^mid^ correspond to cells in S phase, and DAPI^high^ represent cells in G2/M phases.

**Immunoblotting**

For NUP214, DEK and XPO1 immunoblotting, whole cell lysates were prepared with lysis buffer (20 mM HEPES, pH 7.5, 0.4 M NaCl; 1 mM EDTA, 1 mM EGTA, 1 mM DTT) supplemented with a mini complete protease inhibitor cocktail tablet and PhosStop (Roche Diagnostics, Mannheim, Germany), and sonicated two times on ice with 30% amplitude (Sonopuls, Bandelin electronic, Berlin, Germany). Proteins were separated by SDS-PAGE and transferred to a nitrocellulose membrane (ThermoFisher) at 800mA for two hours. Membranes were blocked in 3% non-fat dry milk (Merck, Darmstadt, Germany) for one hour, and incubated overnight at 4°C with the following primary antibodies (anti-NUP214 (A300-716 A, Bethyl laboratories Inc, Montgomery, TX., anti-NUP214 24133-1-AP, Proteintech, Rosemont, IL) (anti-DEK (sc-136222 Santa Cruz Biotechnology, Inc. Dallas, TX), XPO1 (46249 Cell Signaling Technology, Leiden, The Netherlands), anti-beta actin (4970 Cell Signaling Technology), followed by one hour incubation with HRP-conjugated secondary antibodies (Cell Signaling Technology) at room temperature. Chemiluminescence was used for visualization using the ECL Western blotting detection reagents (PerkinElmer, Waltham, MA) according to the manufacturer.

For CRISPR/CAS9 and drug experiments, cells were washed twice with ice-cold PBS and the resulting pellet was resuspended in RIPA buffer, supplemented with Halt™ Protease and EDTA-free Phosphatase Inhibitor Cocktail (100X) (Thermofisher). This suspension was incubated for 1 hour, with slight agitation at 4°C. Lysates were sonicated twice with 30% amplitude (Sonopuls, Bandelin electronic), and cell debris was removed by centrifugation. The supernatant was transferred to new tubes and stored. Proteins were separated by SDS-PAGE and then transferred to a methanol-preactivated PVDF membrane in the Mini Trans-Blot Cell transfer system (Bio-Rad, Feldkirchen, Germany) for 3 hours, using 16 V at 4°C. This was followed by blocking with 3% milk in TBS-T buffer for 1 hour. Membranes were then incubated with anti-XPO1 (Cell Signaling Technologies, and anti-actin (Cell signaling Technologies) antibodies overnight while shaking gently at 4°C. Antibody dilutions are described in Supplemental Table S11. After incubation, membranes were incubated with HRP coupled secondary antibodies for 1 hour at room temperature. Membranes were then incubated with a 1:1 mixture of clarity Max Western ECL substrate detection reagent (Bio-Rad), and the signal was measured with the ChemiDoc System (Bio-Rad).

**Co-Immunoprecipitation (Co-IP)**

Whole cell lysates were prepared from 2x107 FKH-1 cells with lysis buffer (20 mM HEPES, pH 7.5, 0.4 M NaCl; 1 mM EDTA, 1 mM EGTA, 1 mM DTT)  supplemented with mini complete protease inhibitor cocktail (Roche #57350900) and PhosStop (Roche #04906837001). Lysates were sonicated twice with 30% Amplitude for 20 sec and centrifuged at 9000 rpm for 15min at 4°C. Supernatant was collected and stored at -80°C or directly subjected to immunoprecipitation with XPO1 antibody (Cell signaling technologies) pre-coupled to protein G magnetic dynabeads (Invitrogen) at 4°C. After 18 hours, immuno-complexes were recovered, washed, and resuspended in NuPAGE LDS sample buffer (Invitrogen). Samples were boiled for 5 minutes and subjected to western blot analysis.

**Immunofluorescence microscopy (IF)**

After drug treatment, cells were seeded in a 24-well plate containing poly-lysine coated coverslips for at least 2 hours and placed back in the incubator. Cells were washed twice with PBS and fixed with 4% formaldehyde (prepared in PBT buffer) for 20 min. This was followed by three washes with PBT buffer and a blocking step in PBTB for 1 hour at room temperature. Subsequently, cells were incubated with the primary antibody (in PBTB) overnight at 4°C. On the next day, cells were washed three times with PBT buffer and incubated with secondary antibody in PBTB buffer for 1 hour at room temperature. Antibodies are described in Supplemental Table S11. After 2 washes with PBT, DAPI solution was added to the cells. Then the mixture was incubated for 10 minutes at room temperature. A final PBT wash was done, and the coverslips were mounted on clean glass slides using 70% glycerol, and 3 % NPG in PBS. Images were taken on a Axio Imager.M2 (Zeiss, Oberkochen, Germany) using the 40X oil immersion objective. For detection, the excitation filters A647 and A488 were used for XPO1 and NUP214, respectively. Image analysis was carried out with FIJI. [2]

**Assessment of cell morphology**

Cytospin preparations were stained using Wright-Giemsa stain. Morphology was assessed by the Axioscope A1 (Zeiss) microscope with Zeiss immersol and imaged using the Axiocamera 5S. Images were taken with 1000x magnification and processed with the Zen 2.6 lite (blue) software.

**Bioinformatic analysis of RNA-seq data**

Before alignment to the reference genome each sequence in the raw FASTQ files was trimmed on base call quality and sequencing adapter contamination. Reads shorter than 15 bp were removed from the FASTQ files. Trimmed reads were aligned to the human reference genome hg38 with the short read aligner STAR (v.2.4.2a). [3] Read quantification was done with R package “Rsubread” [4] using reference transcriptome GRCh38.79.gtf. [5] Genes with low counts were removed. Gene expression was quantified by CPM (Counts Per Million) values. Data was normalized using the TMM (trimmed mean of M-values) method. [6] Differentially expressed (DE) genes between eltanexor treated and control samples were identified by R package “edgeR” [7] using the multivariate regression model and limma” [8] eBayes functions (Empirical Bayes Statistics for Differential Expression) based on normalized log2(CPM_TMM+1) values. We derived two combinations of samples for differentially expressed genes: treated and control samples in FKH-1 cells (3 vs 3) and treated and control samples in PDX cells (2 vs 2). Heatmaps of differentially expressed genes were generated with R (version 3.4.4, www.r-project.org).

The microarray dataset GSE17855 [9,10] from the GEO repository was processed with R package “affy”, [11] and the expression differences between the samples with and without DEK-NUP214 fusions were visualized by a custom R script. Differences in log2(RPKM) expression values from the Beat AML database [12] were analyzed in Excel. The Broad Institute GSEA software package [13] was employed for gene set enrichment analysis using gene ontology gene sets from the Molecular Signatures Database (http://www.broad.mit.edu/gsea/msigdb/).

**Chromatin immunoprecipitation (ChIP) sequencing**

For chromatin immunoprecipitation, 24 hours post drug treatment, cells were counted and 20 million cells per condition were washed with ice cold PBS and resuspended in fixing buffer (1% Formaldehyde, 100 mM NaCl, 50 mM Hepes-KOH pH 7.5, 1 mM EDTA pH 8.0, 0.5 mM EGTA pH 8.0) for 5 minutes with rotation at RT. All buffers contained protease inhibitors (50 µg/ml PMSF, 1 µg/ ml leupeptin, and 10 M sodium butyrate). The reaction was quenched by adding glycine at a final concentration of 0.125 M for 5 minutes with rotation at RT. The following steps were performed at 4°C. Crosslinked cells were washed with ice cold PBS and resuspended in cell lysis buffer (50 mM Hepes pH 7.9, 140 mM NaCl, 1 mM EDTA, 10% glycerol, 0.25% Triton X-100, 0.5% NP-40) for 10 minutes while rotating. After centrifugation, cells were resuspended in wash buffer (10 mM Tris pH 8.1, 1 mM EDTA pH 8.0, 200 mM NaCl, 0.5 mM EGTA pH 8.0), repeating the incubation step once as before. This was followed by 2 repeats of washing with sonication buffer and resuspension in 1 ml sonication buffer (10 mM Tris pH 8.1, 1 mM EDTA pH 8.0, 0.1% SDS) into a covaris 12 x 12 mm miliTUBE. Cell lysates were sonicated on a covaris S220 ultrasonicator with a 140 peak incident power (PIP), duty factor 5, 200 cycles per burst (CPB) for 12 minutes. After spinning for 10 minutes, supernatant was diluted 1:5 in IP buffer (10 mM Tris pH 8.1, 1 mM EDTA pH 8.0, 150 mM NaCl, 1% Triton X-100, 0.1% SDS) and two preclearing steps were done, first with IgG and then with protein G agarose beads, for 1 hour and 2 hours, respectively. Samples were spun down to remove beads, 2% input removed and incubated with target anti-XPO1 (Cell signaling technology) and anti-NUP214 (Bethyl Laboratories) antibodies overnight with rotation at 4°C.

On the next day, protein G was added to each reaction and incubated for 2 hours. This was followed by washes: twice with a low salt buffer solution (0.1% SDS, 1% Triton X-100, 2mM EDTA, 20mM Tris-HCl pH8.1, 150mM NaCl, twice with a high salt buffer solution (0.1% SDS, 1% Triton X-100, 2mM EDTA, 20mM Tris-HCl pH8.1, 500mM NaCl), and once with TE buffer (10 mM Tris pH 8.0, 1 mM EDTA pH 8.0). Fresh elution buffer (100 mM NaHCO3, 1 % SDS) was added twice and each time samples were vortexed and spun down to reverse crosslinking overnight, together with RNAse (0.02 mg/ml) and sodium chloride (0.3 M) at 67°C in a shaker. Lastly, proteinase K (0.2 mg/ml) was added to samples, which were then incubated at 65°C for 2 hours. DNA was then purified with the Qiagen PCR purification kit and stored at -20°C.

ChIP library preparation and sequencing was performed by Genewiz (Azenta Life Sciences, Leipzig, Germany). Samples were quantified using the Qubit 2.0 fluorometer and the DNA integrity was checked with the 4200 TapeStation (Agilent, Santa Clara, California). The NEB NextUltra DNA Library Preparation kit was used following the manufacturer’s recommendations (Illumina). Briefly, the ChIP DNA was end-repaired and adapters were ligated after adenylation of the 3’ends. Adapter-ligated DNA was size selected, followed by clean up, and limited cycle PCR enrichment. The ChIP library was validated using the Agilent TapeStation and quantified using the Qubit 2.0 Fluorometer as well as real time PCR.

The sequencing libraries were multiplexed and clustered on one lane of a flow cell. Following the clustering process, the flow cell was loaded on the Illumina HiSeq instrument in accordance with the manufacturer’s guidelines (Illumina). Sequencing was performed using a 2x150 Paired-End configuration. Image analysis and base calling were carried out using the HiSeq Control Software (HCS) (Illumina). Raw sequence data (.bcl files), which were generated by Illumina HiSeq, were converted into fastq files and de-multiplexed via Illumina's bcl2fastq 2.17 software. One mismatch was allowed for index sequence identification.

Sequencing adapters and low-quality bases were trimmed using Trimmomatic 0.38. [ref. 14] Cleaned reads were then aligned to the reference genome hg38 using bowtie2 [ref. 15]. Aligned reads were filtered using samtools 1.9 [16] to keep alignments that (1) have a mapping quality ≥30, (2) are aligned concordantly, and (3) are the primary-called alignments. PCR or optical duplicates were marked using Picard 2.18.26 [ref. 17] and removed. Prior to peak calling, reads mapping to mitochondria (mt) were called and filtered and reads mapping to unplaced contigs were removed. MACS2 2.1.2 [ref. 18] was used for peak calling to identify open chromatin regions. If a blacklist of artefactual regions (areas with extremely high or low-mappability) was available for the provided reference genome, called peaks were filtered for these regions to mitigate errors due to mappability. Peak BED files were analyzed with HOMER [19] software: the annotatePeaks.pl script was used to map peak positions to genomic features. Additional visualization was done with the ngs.plot [20] program: density plots of read counts (per million) around genomic regions were plotted in order to show Chip-Seq coverage changes under different conditions. The module chipcor of ChIP-Seq software was used to produce histograms of positional correlation of peaks. Peak pair counts were calculated at 100 bp intervals, presented as smoothed curves and divided by 100 in order to have number of peak pairs within 1 bp interval. Background aggregation levels of histograms under different conditions were matched - then excess aggregation levels could be compared straightforward. ChiP-Seq alignment files were visualized using the Integrative Genomics Viewer (IGV). [21]

**Supplemental Tables**

**Supplemental Table S1.** crRNA sequences.

| **Name** | **Sequence** |
| --- | --- |
| Alt-R® Cas9 Electroporation Enhancer | TTAGCTCTGTTTACGTCCCAGCGGGCATGAGAGTAACAAGAGGGTGTGGTAATATTACGGTACCGAGCACTATCGATACAATATGTGTCATACGGACACG |
| Alt-R® CRISPR-Cas9 Negative Control crRNA #1 | /AltR1/rCrG rUrUrA rArUrC rGrCrG rUrArU rArArU rArCrG rGrUrU rUrUrA rGrArG rCrUrA rUrGrC rU/AltR2/ |
| Alt-R® CRISPR-Cas9 tracrRNA, ATTO™ 550 | AGCAUAGCAAGUUAAAAUAAGGCUAGUCCGUUAUCAACUUGAAAAAGUGGCACCGAGUCGGUGCUUU |
| crRNA 1 | /AltR1/rGrG rUrUrG rArArA rCrCrG rGrUrU rCrArG rArCrU rGrUrU rUrUrA rGrArG rCrUrA rUrGrC rU/AltR2/ |
| crRNA 3 | /AltR1/rUrC rGrArC rUrCrU rUrGrU rCrCrA rArGrC rArUrC rGrUrU rUrUrA rGrArG rCrUrA rUrGrC rU/AltR2/ |
| crRNA 5 | /AltR1/rCrA rGrArU rGrArC rGrUrC rUrUrG rArUrA rArUrG rGrUrU rUrUrA rGrArG rCrUrA rUrGrC rU/AltR2/ |
| crRNA 7 | /AltR1/rGrU rGrArU rArUrU rGrUrU rGrGrA rGrCrA rArGrU rGrUrU rUrUrA rGrArG rCrUrA rUrGrC rU/AltR2/ |
| crRNA 11 | /AltR1/rUrG rArGrU rArCrG rCrArA rGrUrC rGrArA rUrArU rGrUrU rUrUrA rGrArG rCrUrA rUrGrC rU/AltR2/ |

**Supplemental Table S2.** Primer sequences.

| **RT-qPCR** | **Sequence** |
| --- | --- |
| hABL fp | TGGAGATAACACTCTAACCATAACTAAAGGT |
| hABL rp | GATGTAGTTGCTTGGGACCCA |
| DN-F2 rt | AAAGTTGAAGAAACCCCCTACAGA |
| DN-R2 rt | TACTGATGAAGGCGCCGAA |
| SESN1_F2_var1 rt | CGAGTCTTCGGATGGGTTGAAT |
| SESN1_R2_var1 rt | TTCTAATGCCAAGTTCCTGGATG |
| EYA3_F1_var1-3 rt | AGATTTACCAGAGCAACCAGTGA |
| EYA3_R1_var1-3 rt | TGGGAAGGTTTGAAGCAAGG |
| PRDM2_F2_var1-7 rt | GGAGCTCCCCCAAGAGC |
| PRDM2_R2_var1-7 rt | CGTCTTCTTTAGGACCTTCTGC |
| MLL-AF4-Fw [22] | ACAGAAAAAAGTGGCTCCCCG |
| MLL-AF4-Rv [22] | TATTGCTGTCAAAGGAGGCGG |
| ENF207F-MLL-AF4 [23] | CCCAAGTATCCCTGTAAAACAAAAA |
| ENR262R-MLL-AF4 [23] | GAAAGGAAACTTGGATGGCTCA |
| MLL_F [24] | TCCAGAGCAGAGCAAACAGA |
| MLLT3_ex6_R [24] | CTGGGATGGTGTGAAGCTG |
| RUNX1-RUNX1T1-F | CACCTACCACAGAGCCATCAAA |
| RUNX1-RUNX1T1-R | ATCCACAGGTGAGTCTGGCATT |
| XPO1-F1 rt | TGGGAAAACTGAAACCCACCT |
| XPO1-R1 rt | TGCTGGCATAGATTACCAGAGA |
|  |  |
| **PCR/Sanger sequencing** | **Sequence** |
| DEK-FP2 | ACGGAACAGTTCTGGAATGG |
| NUP214-RP2 | GATTTCCCGATTGTTGGCTA |
| 17F [25] | 5′-AATAACCACGACCAGGAATTTG-3′ |
| 17R [25] | 5′-ACGTTTCCACTTCACACACAAG-3′ |
| 20F [25] | 5′-GGCTTCCTCCTTGGATATTTCT-3′ |
| 20R [25] | 5′-CTTTCCGAGGTAGGCCTCTAAC-3′ |

**Supplemental Table S3.** Patient characteristics.

| **Clinical parameters**  **(at diagnosis)** | **#1** | **#2** | **#3** | **#4** | **#5** | **#6** | **#7** | **#8** |
| --- | --- | --- | --- | --- | --- | --- | --- | --- |
| **Diagnosis** | AML | AML | AML | AML | AML | AML | AML | AML |
| **Age** | 19 | 53 | 51 | 58 | 55 | 38 | 28 | 53 |
| **Cytogenetics** | 46,XX,t(6;9) (p23;q34.1) | 46,XX,t(6;9) (p23;q34.1) | 46,XX,t(6;9) (p23;q34.1) | 46,XX,t(6;9) (p23;q34.1) | 46,XY,t(6;9) (p23;q34.1) | 46,XY,t(6;9) (p23;q34.1) | 46,XY,t(6;9) (p23;q34.1) | 46,XY,t(6;9) (p23;q34.1) |
| **White blood cell counts (x10^9^/L)** | 34.1 | 116 | 52.8 | 16 | 19.4 | 13.3 | 4.7 | 25.5 |
| **% Blasts in PB** | 50 | 91 | 29 | 6 | 32 | n.a | 8 | 56 |
| **Hemoglobin at diagnosis (g/dL)** | 6 | 9.4 | 7.4 | 7.3 | 6.4 | 7.7 | 9 | 7.2 |
| **Platelets at diagnosis (x10^9^/L)** | 61 | 38 | 26 | 22 | 72 | 62 | 67 | 59 |
| **% Blasts in BM** | 80 | 94 | 80 | 90 | 10 | 70 | n.a | 85 |
| **Used in PDX** | Yes | Yes | Yes | Yes | Yes | No | No | No |
| **Used in *in vitr*o experiments** | Yes | Yes | Yes | Yes | Yes | Yes | Yes | Yes |

**Supplemental Table S4.** Gene set enrichment analysis of gene expression profiles from FKH-1 eltanexor treated vs FKH-1 control treated cells.

**Supplemental Table S5.** Top500 differentially expressed genes in FKH-1 samples.

**Supplemental Table S6.** Mutations in PDX1 and patient samples.

| **Sample** | **Mutations of patient at diagnosis** | **Mutations**  **at end of 1^st^  Tx** | **Mutations**  **at end of 2^nd^ Tx** | **Mutations**  **at end of 3^rd^ Tx** |
| --- | --- | --- | --- | --- |
| **PDX1** | DEK::NUP214 | DEK::NUP214 | DEK::NUP214 | DEK::NUP214 |
|  | **FLT3-ITD** VAF 33.9% NM_004119.2:c.1731_1793dup,p.Met578_Glu598dup | **FLT3-ITD** VAF 39.0%; NM_004119.2:c.1731_1793dup,p.Met578_Glu598dup | **FLT3-ITD** VAF 42.5%; NM_004119.2:c.1731_1793dup,p.Met578_Glu598dup | **FLT3-ITD** VAF 45.1%; NM_004119.2:c.970G>A,p.Asp324Asn |
|  | **WT1** VAF 35.2%; NM_024426.4:c.1141_1142ins,p.Ser381CysfsTer72; VAF 38.8%; NM_024426.4:c.1132_1133ins,p.Leu378ArgfsTer8 | **WT1** VAF 44.1%; NM_024426.4:c.1141_1142ins,p.Ser381CysfsTer72; VAF 55.2% NM_024426.4:c.1132_1133ins,p.Leu378ArgfsTer8 | **WT1** VAF 44.9%; NM_024426.4:c.1141_1142ins,p.Ser381CysfsTer72; VAF 55.6% NM_024426.4:c.1132_1133ins,p.Leu378ArgfsTer8 | **WT1** VAF 59.8%; NM_024426.4:c.1141_1142ins,p.Ser381CysfsTer72; VAF 42.5% NM_024426.4:c.1132_1133ins,p.Leu378ArgfsTer8 |

Abbreviations: ITD, internal tandem duplication; Tx, transplantation in NSGS mice; VAF, variant allele frequency.

**Supplemental Table S7.** Top500 differentially expressed genes in PDX1 samples.

**Supplemental Table S8.** Gene set enrichment analysis of gene expression profiles from PDX1 eltanexor treated vs PDX1 vehicle treated cells.

**Supplemental Table S9.** Comparison of common upregulated pathways between eltanexor treated PDX1 and FKH-1 cells compared to control treated PDX1 and FKH-1 cells.

**Supplemental Table S10.** Comparison of common downregulated pathways between eltanexor treated PDX1 and FKH-1 cells vs control treated PDX1 and FKH-1 cells.

**Supplemental Table S11.** Antibody information.

| **Target** | **Clone** | **Catalog #** | **Dilution** | **Source** | **Usage** |
| --- | --- | --- | --- | --- | --- |
| CD45-FITC | HI30 | 560976 | 1:50 | BD Biosciences | Flow cytometry |
| CD45-APC/Cy7 | 2D1 | 368516 | 1:50 | Biolegend, San Diego, CA |  |
| CD3-PE | UCHT1 | 555333 | 1:50 | BD Biosciences |  |
| CD3-FITC | Sk7 | 345763 | 1:100 | BD Biosciences |  |
| CD3-APC | Ucht1 | 300412 | 1:50 | Biolegend |  |
| CD14-PE | MΦP9 | 345785 | 1:50 | BD Biosciences |  |
| CD14-APC | Hcd14 | 325608 | 1:50 | Biolegend |  |
| CD14-APC Cy7 | MΦP9 | 557831 | 1:50 | BD Biosciences |  |
| CD15-APC | MMA | 17-0158-42 | 1:50 | Invitrogen, Themofisher |  |
| CD19-PE | HIB19 | 555413 | 1:50 | BD Biosciences |  |
| CD33-APC | WM53 | 983902 | 1:100 | Biolegend |  |
| CD33-APC Cy7 | P67.6 | 366614 | 1:100 | Biolegend |  |
| CD33-PerpCP Cy5.5 | P67.6 | 366616 | 1:66.66 | Biolegend |  |
| CD34-APC | 8G12 | 340441 | 1:100 | BD Biosciences |  |
| CD34-APC Cy7 | 581 | 343514 | 1:66.66 | Biolegend |  |
| CD38-PE | HB7 | 345806 | 1:100 | BD Biosciences |  |
| CD38-FITC | HB7 | 340927 | 1:50 | BD Biosciences |  |
| CD11b-PE | ICRF44 | 555388 | 1:50 | BD Biosciences |  |
|  | D12 | 333142 | 1:50 |  |  |
| Annexin V-APC | - | 550474 | 1:50 | BD Biosciences |  |

| **Target Protein** | **Catalog #** | **Dilution** | **Source** | **Usage** |
| --- | --- | --- | --- | --- |
| Anti-NUP214 rabbit polyclonal | A300-716A | 1:2000  n.a | Bethyl Laboratories | WB,Co-IP, ChIP-seq |
| Anti-XPO1 rabbit polyclonal | ab24189 | 1:500 | Abcam | WB |
| Anti-beta actin rabbit polyclonal | 4967 | 1:2000 or 1:1000 | Cell signaling technology | WB |
| ECL Anti-rabbit IgG horseradish peroxidase linked | NA9340V | 1:5000 | GE Healthcare, Chicago, IL | WB |
| Anti-DEK | sc-136222 | 1:500 | Santacruz | WB |
| Anti-NUP214 rabbit polyclonal | 24113-1-AP | 1:500 | Proteintech | WB |
| Anti-XPO1 mouse monoclonal | 611832 | 1:200 | BD Biosciences | IF |
| Anti-NUP214 rabbit polyclonal | ab70497 | 1:500 | Abcam | IF |
| Goat Alexa 488 anti-rabbit | A-11008 | 1:500 | Invitrogen, Themofisher | IF |
| Goat Alexa 633 anti-mouse | A-21050 | 1:500 | Invitrogen, Thermofisher | IF |
| Anti-XPO1 rabbit monoclonal | 46249 | 1:1000  n.a | Cell signaling technology | WB, Co-IP, ChIP-seq |

**Supplemental Table S12.** Cell lines representative for different genotypes of DEK::NUP214 and FLT3-ITD.

| **DEK::NUP214 negative FLT3-ITD positive** | **DEK::NUP214 negative FTL3-ITD negative** | **DEK::NUP214 positive FLT3-ITD negative** |
| --- | --- | --- |
| MV4-11  MOLM-13 | OCI-AML-2  Kasumi-1 | FKH-1 |

**Supplemental Figures**

**Supplemental Figure S1.** Generation of XPO1 knockout in FKH-1 cells.

1. Confirmation of the DEK-NUP214 fusion gene in FKH-1 cells by Sanger sequencing, where the 5‘end (exon 9) of DEK is fused to the 3‘end (exon 18) of NUP214.
2. Experimental design for electroporation of FKH-1 cells with a CRISPR RNP complex targeting the XPO1 gene. Cells were analyzed starting 24 hours post electroporation by flow cytometry. The targeting regions of the crRNAs are indicated in the transcript of XPO1.
3. Representative phenotype of FKH-1 cells 24 hours post electroporation showing successful CRISPR RNP uptake (percentage of positive tracrRNA-ATTO550 positive cells). Abbreviations: Forward scatter (FSC), control (CTL), non-targeting control (NTC), CRISPR-RNA (cr) 3-5-7, CRISPR-RNA (cr) 1-5-11.
4. Relative mRNA expression of XPO1 in the FKH-1 cell line 96 hours after CRISPR RNP electroporation (mean ± SEM, n=2). Control (CTL), non-targeting control (NTC), CRISPR-RNA (cr) 3-5-7, CRISPR-RNA (cr) 1-5-11.
5. Western blot showing expression of XPO1 in bulk FKH-1 cells 240 hours after electroporation with CRISPR RNPs.
6. Immunofluorescence staining of XPO1 and DEK-NUP214 proteins in FKH-1 cells 240 hours after treatment with mock, NTC (non-targeting control) or anti-XPO1 crRNAs. Z stacks were used to create a maximum intensity projection to show a representative image of the experiments. Experiments were performed twice.


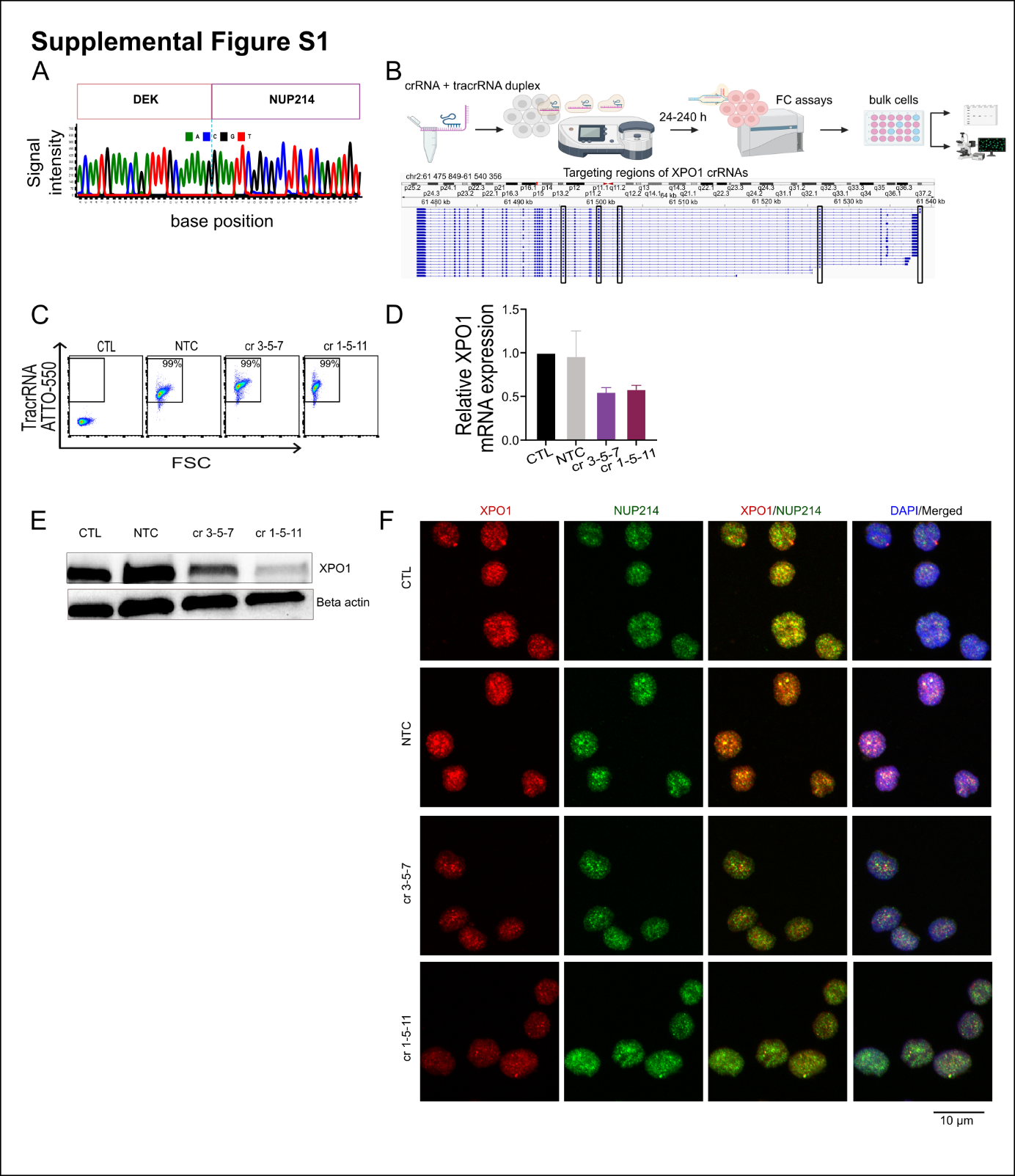


**Supplemental Figure S2.** Biologic effects of XPO1 knockdown in DEK::NUP214 negative cells.

1. Western blot showing expression of XPO1 in bulk OCI-AML2, MV4-11 and Kasumi-1 cells 96 hours after electroporation with CRISPR RNPs.
2. Proportion of annexin V+ apoptotic cells in bulk OCI-AML2, MV4-11 and Kasumi-1 cells after CRISPR RNP electroporation. Non-targeting control (NTC), CRISPR RNA (cr) targeting the exons of XPO1 indicated by the numbers in the figure legend.
3. Viability of bulk OCI-AML2, MV4-11 and Kasumi-1 cells after CRISPR RNP electroporation, assessed as percentage of DAPI negative cells. Non-targeting control (NTC), CRISPR RNA (cr) targeting the exons of XPO1 indicated by the numbers in the figure legend.

**
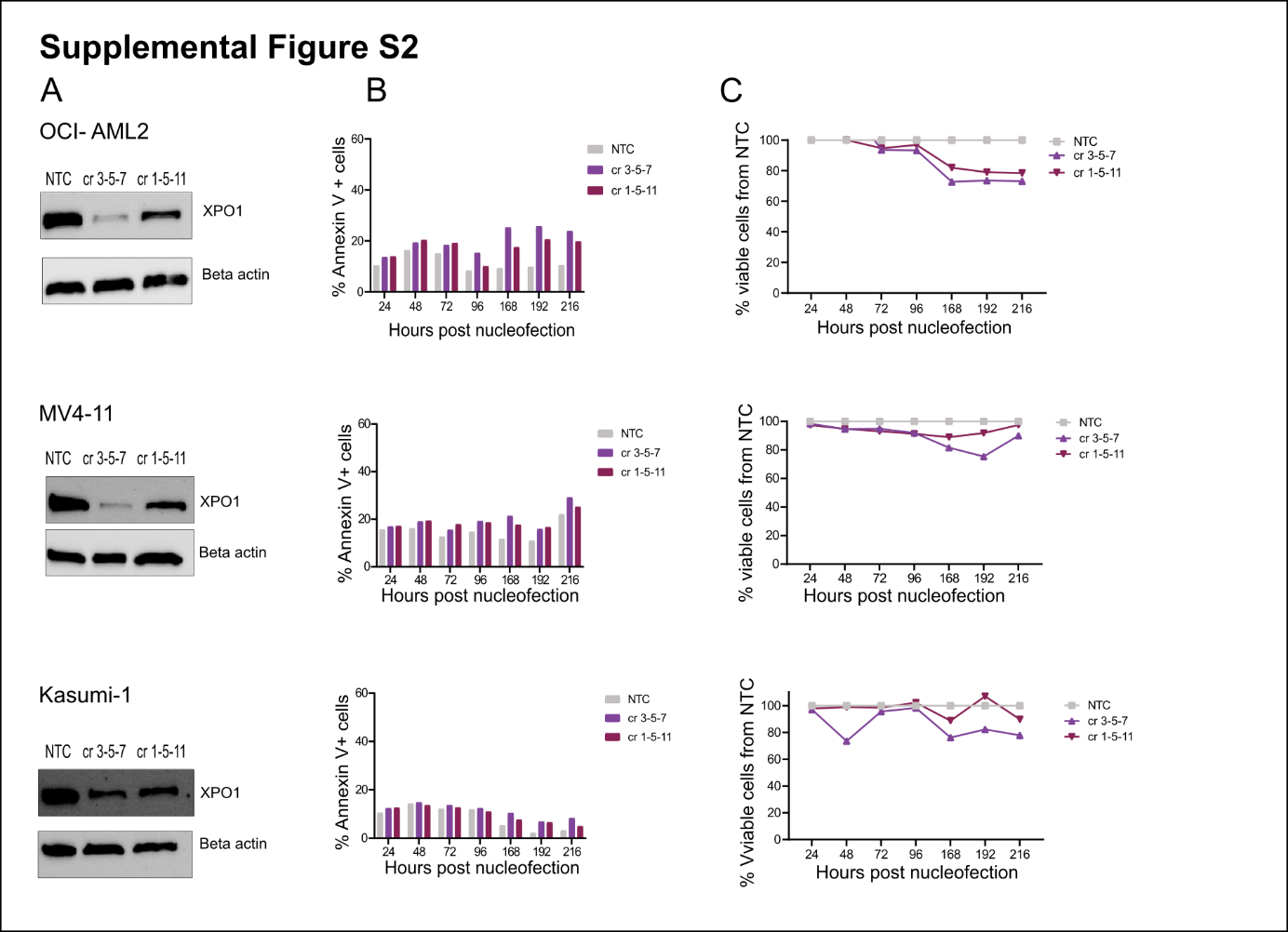
**

**Supplemental Figure S3.** XPO1 inhibition in vitro by eltanexor.

1. Western blot showing expression of XPO1 in eltanexor and control treated FKH-1 cells at 24 hours.
2. Immunofluorescence confocal microscopy of FKH-1 cells after 24 hours of treatment with eltanexor at a dose of 400 nM. Z stacks were used to create a maximum intensity projection to show a representative image of the experiments. A representative image is shown from experiments performed in triplicates.

Statistically significant results are marked with an asterisk. *p<0.05, **p<0.01, ***p<0.001.

**
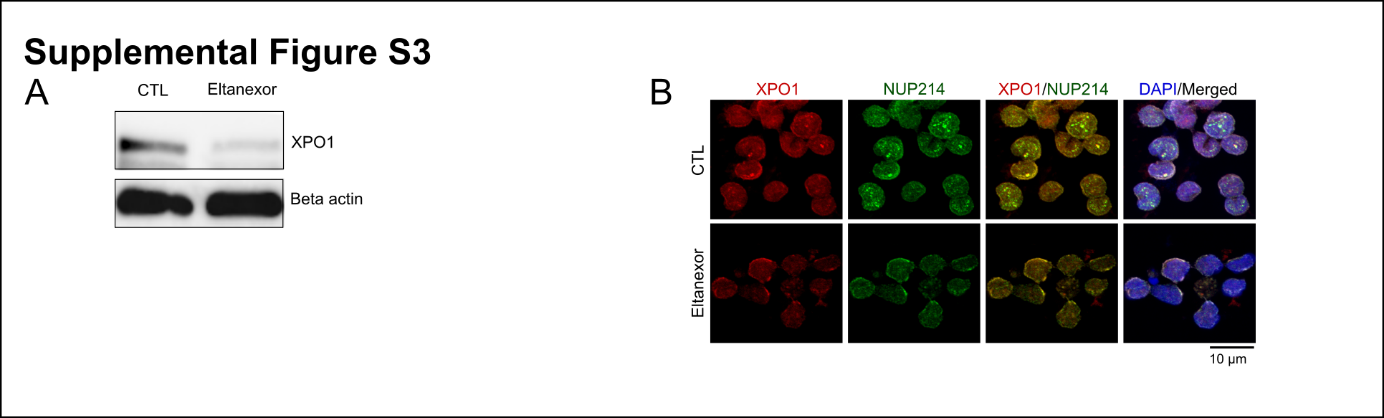
**

**Supplemental Figure S4.** XPO1 inhibition in vitro by selinexor.

1. Cell viability of FKH-1 cells after 96 hours of treatment with the indicated concentrations of selinexor (mean ± SEM, n=3).
2. Frequency of annexin V+ apoptotic FKH-1 cells after 96 hours of treatment with the indicated concentrations of selinexor (mean ± SEM, n=3).
3. Changes in gene expression of *DEK::NUP214* putative target genes in FKH-1 cells after 96 hours of treatment with selinexor at the indicated concentrations (mean ± SEM, n=3).

Statistically significant results are marked with an asterisk. *p<0.05, **p<0.01, ***p<0.001.

**
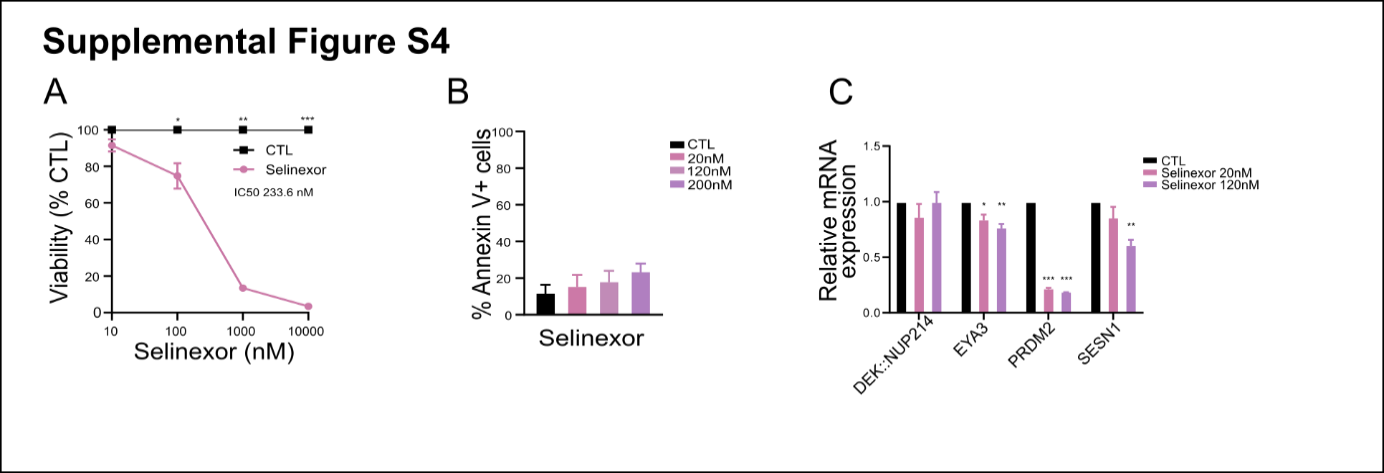
**

**Supplemental Figure S5.** XPO1 inhibition in DEK::NUP214 patient samples by eltanexor.

1. Viability of DEK::NUP214 positive patient samples three days after eltanexor treatment (mean ± SEM, CTL=8 (samples #1-8); 40 nM n=7 (samples #1-6, 8); 200 nM =8 (samples #1-8); 1000 n=6 (samples #1-3, 5, 6, 8).
2. Representative immunofluorescence image of bone marrow cells from patient 1 after 72 hours of in vitro treatment with eltanexor at a dose of 200 nM. Z stacks were used to create a maximum intensity projection image. Sample #1

**
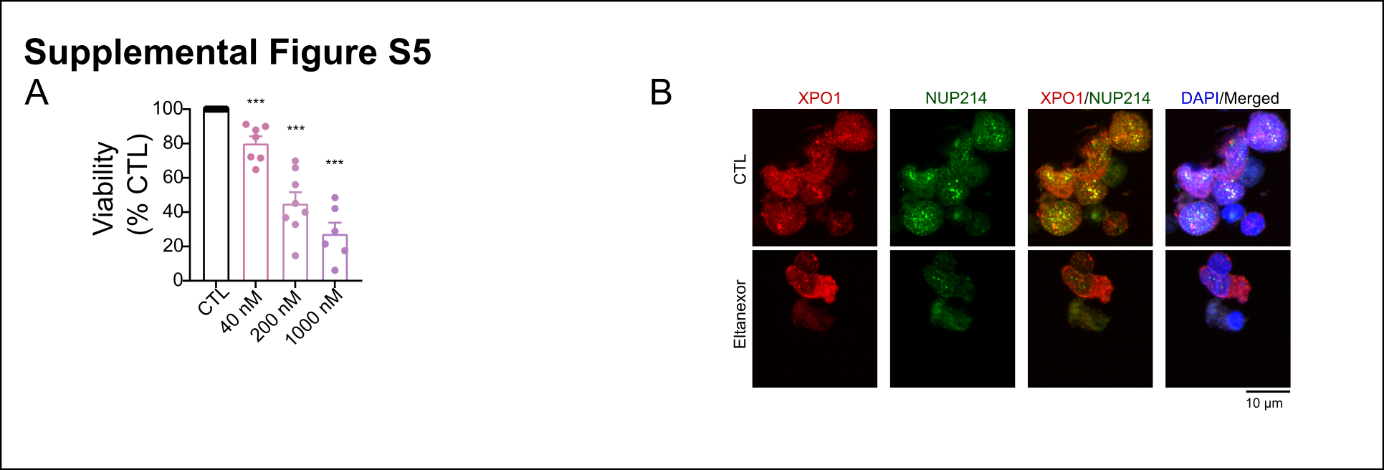
**

**Supplemental Figure S6.** Eltanexor reduces co-localization of XPO1 and NUP214 at the promoter of DEK::NUP214 target genes.

A. ChIP-Seq tracks for XPO1 (top) and NUP214 (bottom) in FKH-1 cells treated with DMSO (black) or eltanexor at a dose of 400 nM for 24 hours (purple). The genomic loci of the DEK-NUP214 putative target genes *EYA3*, *PRDM2* and *SESN1* are shown in the bottom rows. The highlighted areas show the promoter region of the different transcripts. Input is shown in gray as control.

**
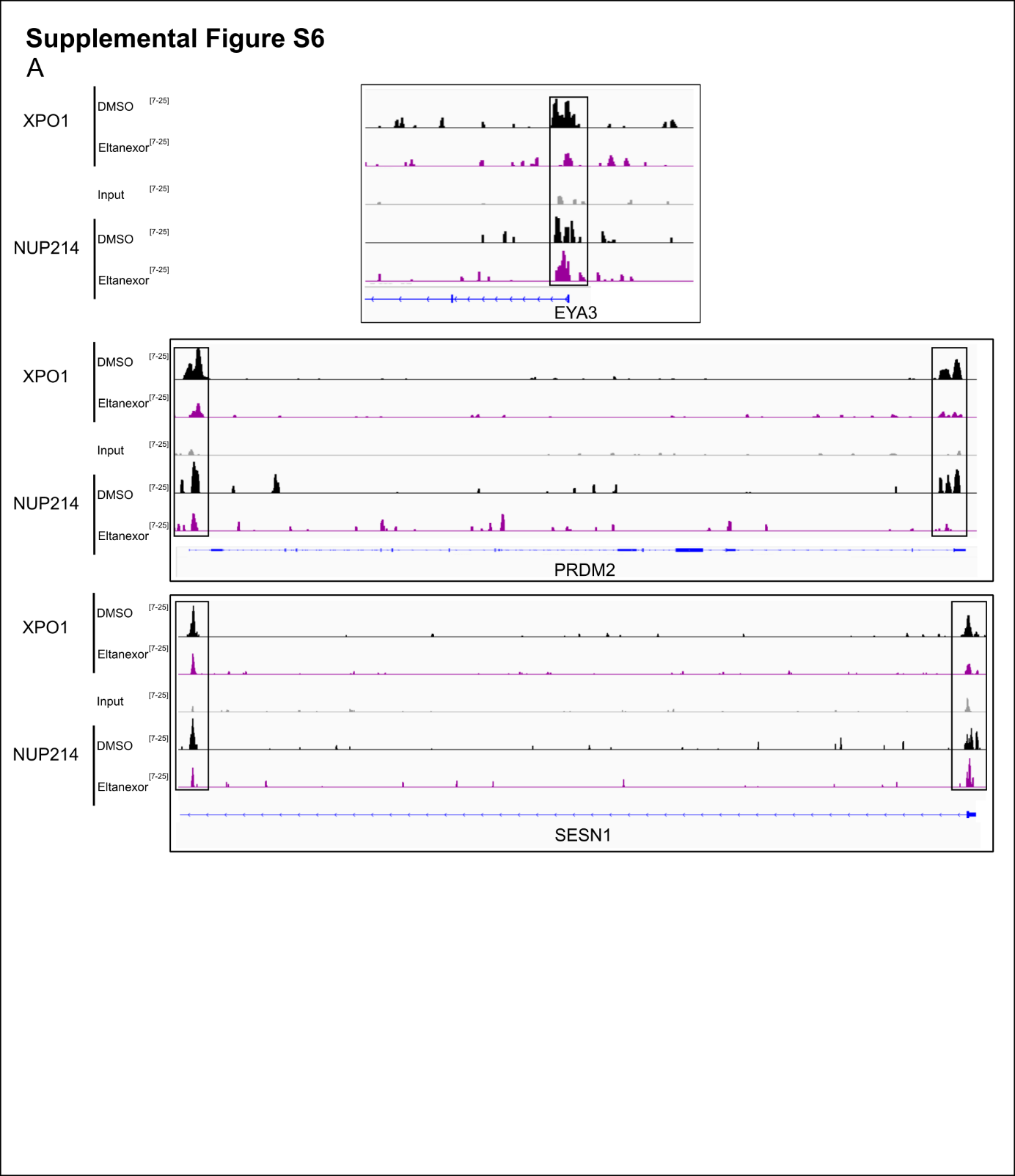
**

**Supplemental Figure S7.** Transcriptional effects of XPO1 inhibition by eltanexor in vitro and in vivo.

1. Immunophenotype of bone marrow and spleen cells from vehicle and eltanexor treated mice at time of sacrifice (mean ± SEM, n=5, for vehicle, =4, for eltanexor).
2. PCA analysis of FKH-1 cells and PDX1 cells following treatment with eltanexor or control. Abbreviations: cell line-control (CL-C), cell line-eltanexor (CL-E), PDX1-control (PDX-C), PDX1-eltanexor (PDX-E)
3. Gene set enrichment analysis for PDX cells treated with 12.5 mg/kg of eltanexor for 11 days or solvent control based KEGG pathways.

**
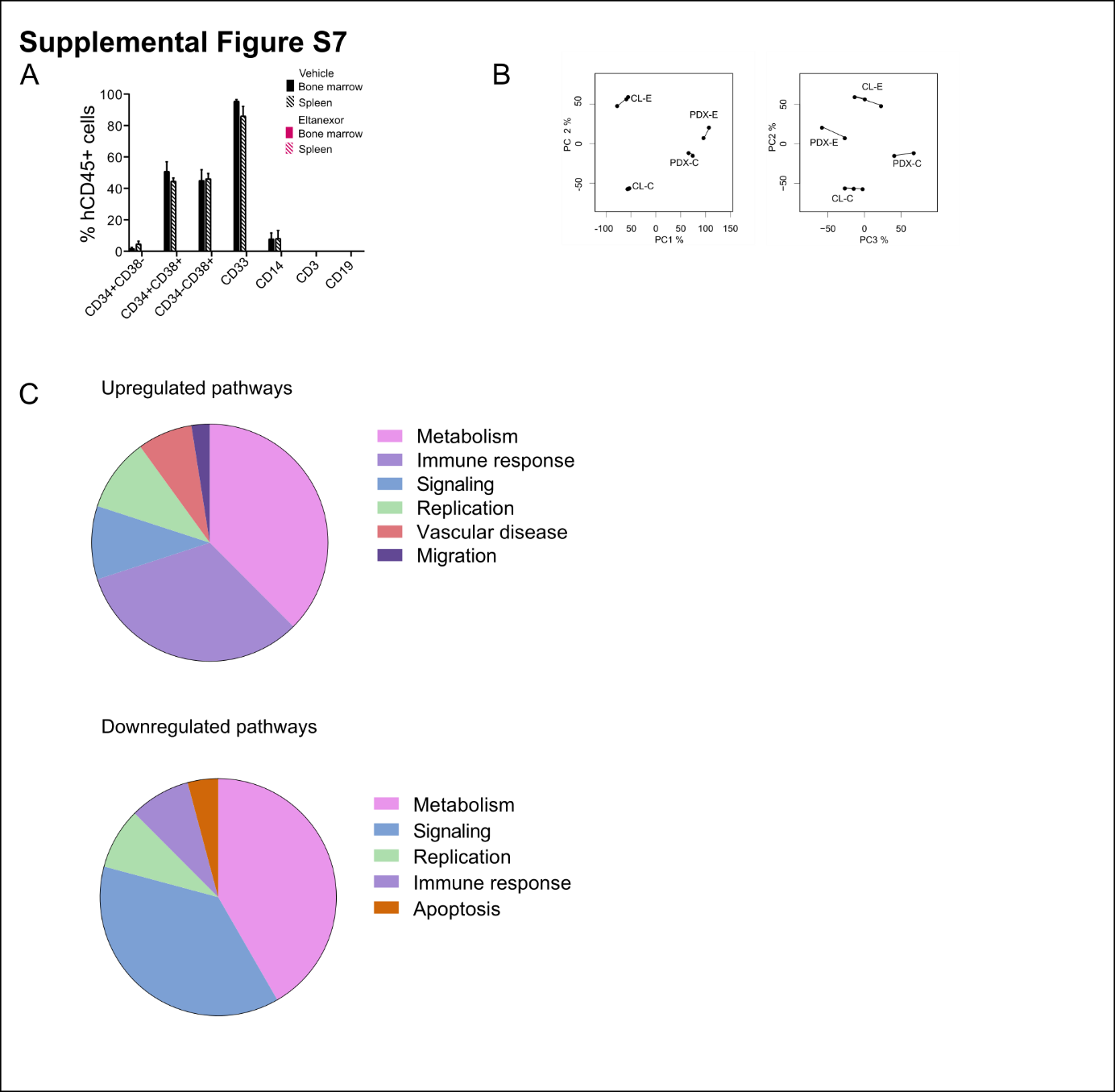
**

**Supplemental Figure S8.** Combination treatment of several cell lines with eltanexor and gilteritinib to evaluate potential synergy.

1. Combination index and fraction affected (Fa) in MV4-11 and MOLM-13 cells
2. Combination index and fraction affected (Fa) in OCI-AML2 and Kasumi-1 cells
3. Combination index and fraction affected (Fa) in FKH-1 cells.

Fa 0 = lack of effect to 1 = maximal effect. Data from three independent experiments for each drug combination are shown.

**
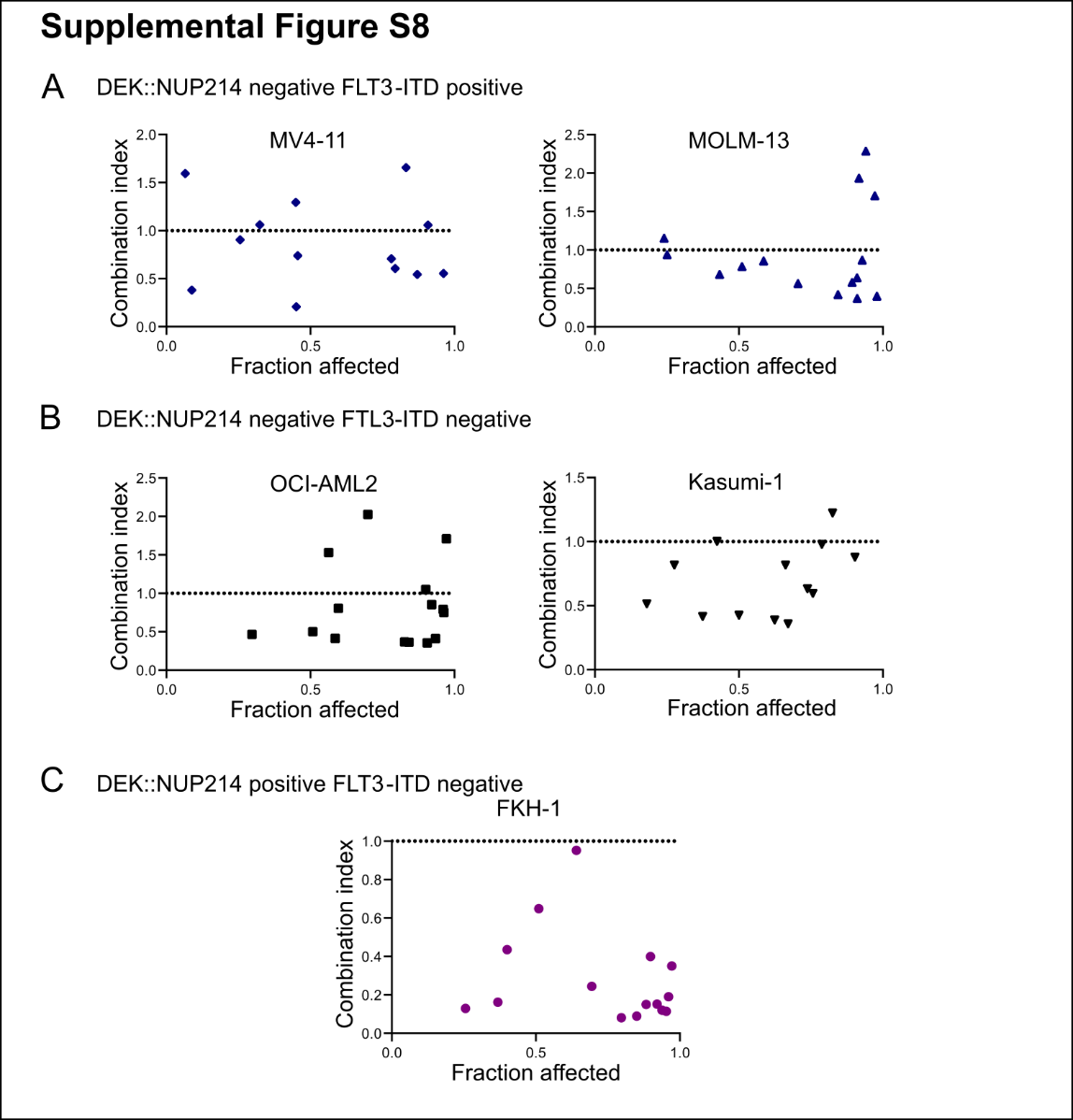
**

**Supplemental references**

1. Rausch T, Fritz MHY, Untergasser A, Benes V. Tracy: basecalling, alignment, assembly and deconvolution of sanger chromatogram trace files. BMC Genomics. 2020 Dec 14;21(1):230.

2. Schindelin J, Arganda-Carreras I, Frise E, Kaynig V, Longair M, Pietzsch T, et al. Fiji: An open-source platform for biological-image analysis. Nat Methods. 2012;9(7):676–82.

3. Dobin A, Davis CA, Schlesinger F, Drenkow J, Zaleski C, Jha S, et al. STAR: ultrafast universal RNA-seq aligner. Bioinformatics. 2013;29(1):15–21.

4. Liao Y, Smyth GK, Shi W. The Subread aligner: fast, accurate and scalable read mapping by seed-and-vote. Nucleic Acids Res. 2013 May 1;41(10):e108–e108.

5. Harrow J, Frankish A, Gonzalez JM, Tapanari E, Diekhans M, Kokocinski F, et al. GENCODE: The reference human genome annotation for The ENCODE Project. Genome Res. 2012 Sep;22(9):1760.

6. Robinson MD, Oshlack A. A scaling normalization method for differential expression analysis of RNA-seq data. Genome Biol. 2010;11(3):1–9.

7. Robinson MD, McCarthy DJ, Smyth GK. edgeR: a Bioconductor package for differential expression analysis of digital gene expression data. Bioinformatics. 2010 Jan 1;26(1):139.

8. Ritchie ME, Phipson B, Wu D, Hu Y, Law CW, Shi W, et al. limma powers differential expression analyses for RNA-sequencing and microarray studies. Nucleic Acids Res. 2015 Apr 20;43(7):e47–e47.

9. Balgobind B V., van den Heuvel-Eibrink MM, De Menezes RX, Reinhardt D, Hollink IHI, Arentsen-Peters STJCM, et al. Evaluation of gene expression signatures predictive of cytogenetic and molecular subtypes of pediatric acute myeloid leukemia. Haematologica. 2011 Feb 1;96(2):221–30.

10. Sandahl JD, Coenen EA, Forestier E, Harbott J, Johansson B, Kerndrup G, et al. T(6;9)(p22;q34)/DEK-NUP214-rearranged pediatric myeloid leukemia: An international study of 62 patients. Haematologica. 2014 May 1;99(5):865–72.

11. Gautier L, Cope L, Bolstad BM, Irizarry RA. affy—analysis of Affymetrix GeneChip data at the probe level. Bioinformatics. 2004 Feb 12;20(3):307–15.

12. Tyner JW, Tognon CE, Bottomly D, Wilmot B, Kurtz SE, Savage SL, et al. Functional genomic landscape of acute myeloid leukaemia. Nature. 2018;562(7728):526–31.

13. Subramanian A, Tamayo P, Mootha VK, Mukherjee S, Ebert BL, Gillette MA, et al. Gene set enrichment analysis: A knowledge-based approach for interpreting genome-wide expression profiles. Proc Natl Acad Sci U S A. 2005 Oct 25;102(43):15545–50.

14. Bolger AM, Lohse M, Usadel B. Trimmomatic: a flexible trimmer for Illumina sequence data. Bioinformatics. 2014 Aug 1;30(15):2114–20.

15. Langmead B, Salzberg SL. Fast gapped-read alignment with Bowtie 2. Nat Methods 2012 94. 2012 Mar 4;9(4):357–9.

16. Danecek P, Bonfield JK, Liddle J, Marshall J, Ohan V, Pollard MO, et al. Twelve years of SAMtools and BCFtools. Gigascience. 2021 Feb 1;10(2):1–4.

17. Institute B. A set of command line tools (in Java) for manipulating high-throughput sequencing (HTS) data and formats such as SAM/BAM/CRAM and VCF [Internet]. Available from: http://broadinstitute.github.io/picard/

18. Liu T. Use Model-Based Analysis of ChIP-Seq (MACS) to Analyze Short Reads Generated by Sequencing Protein--DNA Interactions in Embryonic Stem Cells. In: Kidder BL, editor. Stem Cell Transcriptional Networks: Methods and Protocols. New York, NY: Springer New York; 2014. p. 81–95.

19. Heinz S, Benner C, Spann N, Bertolino E, Lin YC, Laslo P, et al. Simple combinations of lineage-determining transcription factors prime cis-regulatory elements required for macrophage and B cell identities. Mol Cell. 2010 May 5;38(4):576.

20. Shen L, Shao N, Liu X, Nestler E. Ngs.plot: Quick mining and visualization of next-generation sequencing data by integrating genomic databases. BMC Genomics. 2014 Apr 15;15(1):1–14.

21. Robinson JT, Thorvaldsdóttir H, Winckler W, Guttman M, Lander ES, Getz G, et al. Integrative Genome Viewer. Nat Biotechnol. 2011;29(1):24–6.

22. Thomas M, Geßner A, Vornlocher HP, Hadwiger P, Greil J, Heidenreich O. Targeting MLL-AF4 with short interfering RNAs inhibits clonogenicity and engraftment of t(4;11)-positive human leukemic cells. Blood. 2005;106(10):3559–66.

23. Gabert J, Beillard E, van der Velden VHJ, Bi W, Grimwade D, Pallisgaard N, et al. Standardization and quality control studies of ‘real-time’ quantitative reverse transcriptase polymerase chain reaction of fusion gene transcripts for residual disease detection in leukemia – A Europe Against Cancer Program. Leukemia. 2003 Dec 9;17(12):2318–57.

24. Løvf M, Thomassen GOS, Bakken AC, Celestino R, Fioretos T, Lind GE, et al. Fusion gene microarray reveals cancer type‐specificity among fusion genes. Genes, Chromosom Cancer. 2011 May 8;50(5):348–57.

25. Tiacci E, Spanhol-Rosseto A, Martelli MP, Pasqualucci L, Quentmeier H, Grossmann V, et al. The NPM1 wild-type OCI-AML2 and the NPM1-mutated OCI-AML3 cell lines carry DNMT3A mutations. Leukemia. 2012;26(3):554–7.
